# A protocol for the integration of multi-omics bioinformatics: Mechanism of acupuncture as an adjunctive therapy for alcohol use disorder

**DOI:** 10.3389/fneur.2022.977487

**Published:** 2023-01-05

**Authors:** Peiming Zhang, Xiaochang Lan, Baochao Fan, Yiming Chen, Xiaojing Wei, Xiangli Li, Ni Fan, Chunzhi Tang, Liming Lu

**Affiliations:** ^1^Clinical Research and Big Data Laboratory, South China Research Center for Acupuncture and Moxibustion, Medical College of Acu-Moxi and Rehabilitation, Guangzhou University of Chinese Medicine, Guangzhou, Guangdong Province, China; ^2^Department of Substance Dependence, The Affiliated Brain Hospital of Guangzhou Medical University (Guangzhou Huiai Hospital), Guangzhou, Guangdong Province, China; ^3^Department of Chinese Medicine, Guangzhou First People's Hospital, Guangzhou, Guangdong Province, China

**Keywords:** alcohol use disorder, Jin's three-needle technique, acupuncture, microbiome, metabolomics, multi-omics, multi-modal bioinfomation

## Abstract

**Background:**

Alcohol use disorder (AUD) has become a significant global factor in various diseases. As a non-pharmacological therapy, certain therapeutic potential has been found in acupuncture; however, in-depth mechanistic studies related to acupuncture for patients with AUD are still insufficient.

**Methods:**

Based on a randomized control design and a multi-omics analysis plan, this protocol details the recruitment (42 AUD patients), group allocation (21 in acupuncture group vs. 21 in sham acupuncture group), intervention and follow-up (replacement drugs as a normal treatment, 2 weeks acupuncture duration, and 3 month follow-up), and data collection and analytical processes. For the clinical outcomes, in addition to the time required for alcohol withdrawal symptoms to subside as the primary outcome, changes in the alcohol withdrawal symptoms, alcohol craving, mood dysfunction, sleep disorder, fatigue, self-efficacy, gastrointestinal symptoms, the quality of life, and the relapse outcomes will be compared between the groups to confirm the acupuncture clinical effectiveness on alcohol withdraw. The gut microbiome and the fecal metabolomics will also be assessed to explore the association of the structure and the function of gut microflora and the mediation of acupuncture effect on AUD fully utilizing gut microflora multi-modal data and clinical information, via the combination of multi-omics methods, feature screening algorithms and appropriate models.

**Discussion:**

The results of this study may help to strengthen clinical evidence of the mechanism of acupuncture intervention in patients with AUD, through understanding of the regulatory mechanism of acupuncture in the gut microbiome and its metabolism as well as AUD-related clinical manifestations.

**Trial registration:**

Chinese Clinical Trial Registry ChiCTR2200058120. Registered on 24 Mar 2022.

## 1. Introduction

Alcohol use disorder has become a significant global factor in various diseases (1–4). In recent years, the health benefits in many developing countries and regions have been impaired by increased alcohol consumption (3–6). Alcohol use disorder (AUD) is one of the most prevalent chronic relapsing substance use disorders (SUDs), with widespread negative impacts on global public health (7).

As a systematic mechanism of bidirectional information exchange between the brain and the gut microbiota (8), the microbiota-gut-brain axis has received significant attention, with an increasing number of findings suggesting that the gut microbiota and drinking-related behavioral factors can interact with each other (9–11). It has been clinically observed that many patients with AUDs (especially alcohol-dependent patients) often present obvious gastrointestinal symptoms in the early stages of abstinence, as well as alcohol-related psychiatric symptoms. Gastrointestinal symptoms are some of the most common somatic symptoms in alcohol withdrawal syndrome (AWS), often gradually relieved with the decline of psychiatric symptoms. On the one hand, alcoholism may lead to a decrease in the protective gut microbiome or an increase in the pathogenic gut microbiome, as well as bacterial translocation. A recent systematic review (12) showed that alcoholism leads to a disease-specific alteration in the gut microbiota, with a lower relative abundance of phylum bacteroidetes, most firmicutes, and actinobacteria (especially the genus Bilobacteria) in alcoholic individuals, but a higher relative abundance of enterobacteriaceae and the genus streptococcus, characterized by reduced obligate anaerobic bacteria and increased facultative anaerobic bacteria. Such alterations may involve potential mechanisms or hypotheses such as down-regulation of antimicrobial peptides, ethanol-induced oxidative stress, alterations in bile salt concentrations (13), bacterial overgrowth due to slowing of bowel motility, increased fecal pH (14), and interference in bacteriocin secretion by some enterobacteria which affect other bacteria (15). On the other hand, alterations in gut microbiota and its metabolism fundamentally influence brain function through many potential pathways such as the nervous and immune systems (16–19), involving many types of signaling such as enteroendocrine, neurotransmitters (20–22), branched-chain amino acids, and short-chain fatty acids (23). Previous studies have suggested that alterations of gut microbes can generate effects on specific brain regions, involving myelin plasticity (24, 25), neuroinflammatory response (26, 27), and neurotransmitter function (28, 29) which mediate related behavioral changes. However, microbiome-gut-brain interactions are still not clear in patients with AUD, and the microorganisms and their metabolites that are sensitive to AUD-specific interventions remain unexplored.

The efficacy of acupuncture on patients with SUDs has been verified by long-term clinical observations; acupuncture helps to alleviate pathological cravings, promote regression of acute withdrawal syndrome, reduce protracted discomfort of withdrawal, decrease relapse, and improve social function and quality of life (30, 31). A previous study with a sample size of 503 patients revealed that nearly half of the subjects reported a decrease in alcohol desire after acupuncture intervention (32). As previous laboratory studies have also confirmed, acupuncture stimulation can alleviate stress and alcohol-related anxiety (33), relieve pain during alcohol withdrawal (34), decrease alcohol dependence (35), enhance self-control (36), and improve learning and memory functions (33, 37–40). Previous molecular-level studies have shown that acupuncture can rebalance AUD-induced maladaptation of neurotransmitters and hormones in related brain regions, inhibit the inflammatory response of the central nervous system (CNS), and protect the function of important brain regions (35, 39, 41–45).

Many studies have indicated that acupuncture can improve brain function and behavior through regulation of the microbiome state. For example, acupuncture can alleviate behavioral deficits by modulating gut microbiota, neuroinflammation, and brain-gut peptide expression in mice suffering from Parkinson's disease (46, 47). Acupuncture can also exert antidepressant effects by modulating the gut microbiota and neurotransmitters in depressive rats (48). At present, studies have shown that acupuncture not only restores the relative abundance of gut microbial genera in mice, but also restores physiological functions such as glutathione metabolism, methane metabolism, and Parkinson's disease (PD) pathways, and improves their motor function and comorbid anxiety (49). In addition, studies have shown that acupuncture inhibits the inflammatory response of CNS by balancing the gut microbial structure, reducing inflammatory factors (such as lipopolysaccharide, interleukin-1β (IL-1β), and IL-6), and inhibiting inflammatory pathways (50). Thus, acupuncture can mediate the improvement of gut microbial structure and function, regulating psychiatric symptoms. In other words, gut microbial information may provide improved efficacy and prognostic indications for the clinical application of acupuncture in patients with AUD. However, previous studies on acupuncture in patients with AUD mainly evaluated the efficacy, but seldom explored the mechanism; a number of articles have introduced laboratory indicators; however, most of these indicators were used to assess alcohol-related psychiatric symptoms as their biomarker (e.g., serum homocysteine (Hcy) for cognitive function) or their objective reflection (e.g., event-related potentials (ERPs) and exploratory eye movement (EEM) for cognitive function) (51). Thus, acupuncture has a co-regulatory effect on the brain and the gut, but still requires further observation in patients with AUD.

In summary, pre-evidence based (49, 52) acupuncture may help improve AUD by regulating the gut microbiome and its metabolism. Moreover, the fact that the fecal metabolome can be used as a functional readout of the gut microbiome (53) supports a combination of these omics tools to obtain multimodal information on the gut microbial communities. Therefore, the gut microbiome and the fecal metabolomics of patients with AUD, and their role in the effect of acupuncture on the psychopathology of patients with AUD will be explored in this study.

## 2. Study aims

The purpose of this study is to report a protocol for the integration of multi-omics bioinformatics and the mechanism of acupuncture as adjunctive therapy for AUD, including three sub-studies:

**(1) The clinical psychiatric symptom sub-study** aims to evaluate the clinical symptom response to acupuncture in patients with AUD.

**(2) The intestinal microbiome and metabolomics sub-study** aims to compare changes in the biodiversity and metabolites between pre- and the post-intervention in both groups of patients.

**(3) The joint analysis sub-study** aims to demonstrate associations between the gut microbiome and the clinical changes of patients with AUD after acupuncture intervention, identifying important factors affecting the response to acupuncture and assessing their contribution to the impact.

## 3. Design and methodology

### 3.1. Design

**Figure 1:**
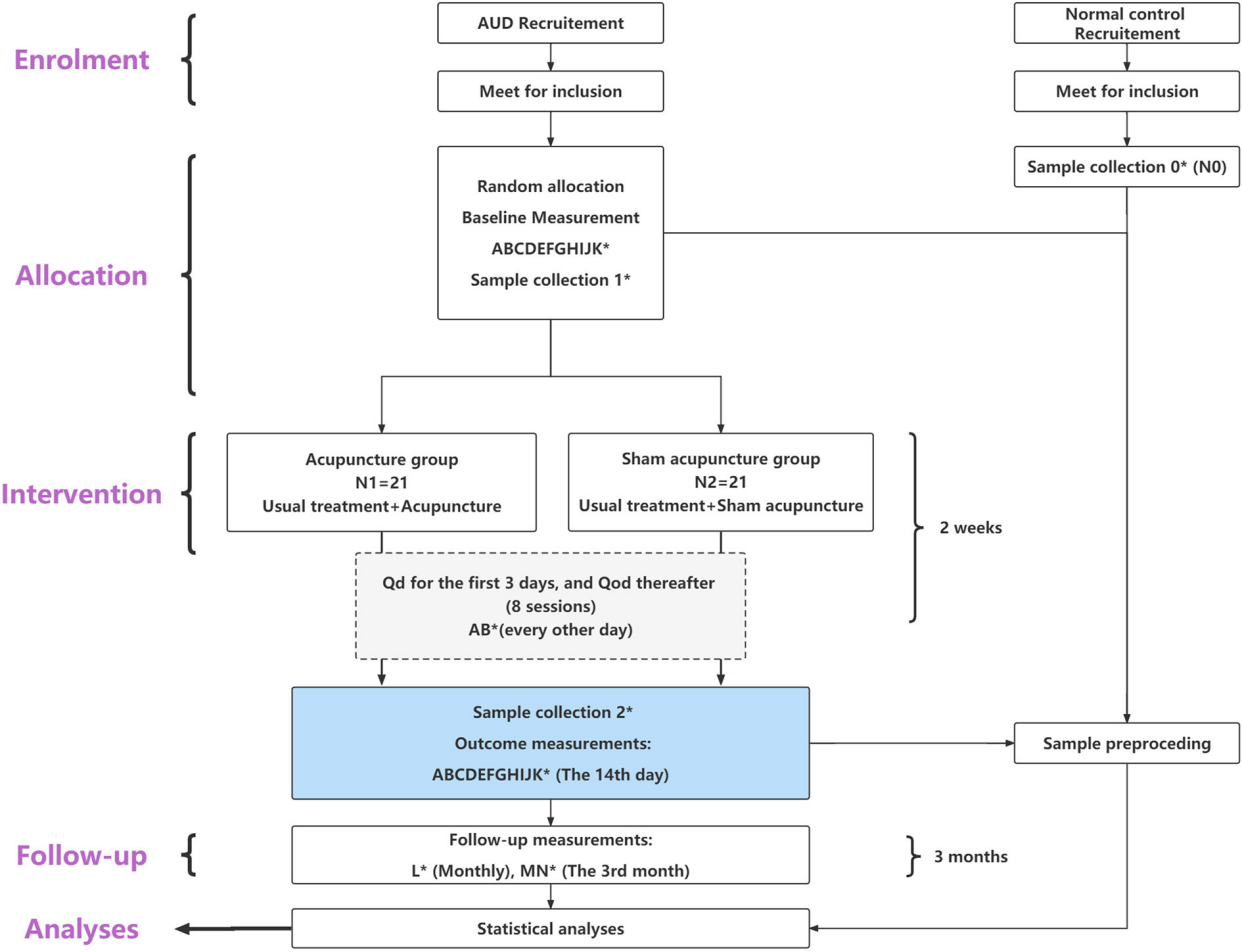
Flow diagram of the study process. ^*^Sample collection 0, 1, 2 means to collect 10 fecal samples from each patient group (N1 and N2) at baseline and at the end of the last intervention; additionally, to collect 10 fecal samples from the health control group (N0). (A–N) means the 14 measurement tools applied in this study, distributed in specific study phases. (A) CIWA-Ar, Clinical institute alcohol withdrawal syndrome scale. (B) abs-VAS, Abstinence visual analog-rating scale. (C) AUQ, Alcohol urge questionnaire. (D) cue-VAS, Cue-related visual analog-rating scale. (E) BDI-II, Beck depression inventory II. (F) BAI, Beck anxiety inventory. (G) PSQI, Pittsburgh sleep quality index. (H) MFS, Mental fatigue scale. (I) GSES, General self-efficacy scale. (J) Gastrointestinal symptom rating scale. (K) QOL-DA, Quality of life scale for patients with drug addiction/dependence. (L) TLFB, Timeline followback. (M) AUDIT, Alcohol use disorder identification test. (N) MAST, Michigan alcohol screening test.

The trial is a randomized, placebo-controlled, 2-arm parallel-group clinical trial, with participants randomized to receive acupuncture or sham acupuncture over 2 weeks. The study was ethically approved by the Institutional Review Board of the Affiliated Brain Hospital of Guangzhou Medical University (ABHGMU, project number 2022/007). The trial has been registered with the Chinese Clinical Trial Registry (ChiCTR2200058120). This study protocol conforms to the Standard Protocol Items: Recommendations for Interventional Trials (SPIRIT) guidelines (see Section 1, Supplementary Table S1 in Appendix 1) (54) and the Standards for Reporting Interventions in Controlled Trials of Acupuncture (STRICTA) checklist (see Section 2, Supplementary Table S2 in Appendix 1) (55). The flowchart of the study is shown in Figure 1.

### 3.2. Center

This trial was designed by the Clinical Research and Big Data Laboratory, South China Research Center for Acupuncture and Moxibustion, Medical College of Acu-Moxi and Rehabilitation, Guangzhou University of Chinese Medicine (GZUCM). Clinical data collection is currently being conducted at ABHGMU, Guangzhou, China.

### 3.3. Participants

Enrollment for the study began on April 1 2022, with an expected completion date of March 31 2023. It is expected that 42 participants from the overall sample will be recruited into the patient group. Based on cultural factors and features of the population of China, the target population of this study is male patients with AUD. The inclusion and exclusion criteria for eligibility are as follows:

### 3.4. Inclusion

(1) In line with the diagnosis of AUD in the American Psychiatric Association's Diagnostic and Statistical Manual of Mental Disorders, Fifth Edition (DSM-V) (see Section 4, Supplementary Table S3 in Appendix 1) (56).
(2) Male patients.
(3) Aged between 18–70 years old.
(4) Proposed or ongoing systematic management of alcohol withdrawal.
(5) Understand the study and sign the informed consent.

### 3.5. Exclusion

Subjects will be excluded if they meet any of the following criteria:

(1) Patients with any serious neurological or psychiatric diseases caused by diseases other than alcohol dependence (including brain tissue damage caused by traumatic brain injury).
(2) Use of other psychoactive substances apart from alcohol, including traditional drugs and new psychoactive substances, or smoking over 30 cigarettes per day.
(3) Patients with severe disease of the heart, liver, spleen, lung, or kidney.
(4) Patients with syphilis or acquired immunodeficiency syndrome (AIDS).
(5) Those with severe digestive system diseases or severe malnutrition.
(6) Patients with severe primary diseases of the hematopoietic system.
(7) Patients with abnormal coagulation function.
(8) Those with inflammation, scars, or trauma at the operation site, or those with severe systemic infection.
(9) Those with cognitive dysfunction, those unable to cooperate, or receiving other treatments that may affect assessment.
(10) Those who have received any acupuncture treatment in the past 6 months.

### 3.6. Sample size calculation

As the primary outcome of this study, the time required for the alcohol withdrawal reaction to subside is 11 ± 5 days in the routine intervention group and 5 ± 3 days in the acupuncture adjunct intervention group, with a minimal clinically important difference (MCID) of 1.5, based on our previous clinical professional experience. Under the one-sided condition α = 0.025 and test power 1–β = 0.90, at least 19 cases are required in each group, based on estimation using the superiority assumption in PASS 11. Given that all subjects will be recruited from inpatients, and two of the most common reasons for dropout are early discharge and transfer to different departments, taking a dropout rate of 10% into consideration, the sample size in each group is: 19/(1–10%) = 21 cases, giving a total of 42 cases. Considering that there has not been relevant research on acupuncture intervention in patients with AUD, and assessment of the intestinal microbiome, this study is a preliminary exploration. Referring to a number of similar studies (57–61), data collection of intestinal microbiome and metabolomics will be performed for at least 10 cases in each patient group. Additionally, 10 fecal samples from both healthy controls [teetotalers or light social drinkers, with an Alcohol Use Disorder Identification Test (AUDIT) score ≤ 7, and normal cognition and memory function assessed using the Mini-mental State Examination (MMSE)] and AUD participants will be collected to determine differences in gut microbiomes and metabolomics between AUD and normal groups.

### 3.7. Randomization procedure

Before randomization, all eligible participants will be asked to sign an informed consent (the index in Section 5 in Appendix 1, and the operative document in Appendix 2). A random sequence in a block size of four will be generated by the central random allocation system developed by the Clinical Research and Big Data Laboratory of GZUCM. Participants will be informed that they have an equal chance of being assigned to the acupuncture or control group. Eligible patients (*N* = 42) will be assigned a random number by an independent researcher who is not involved in the outcome assessment, and will be randomly allocated to the acupuncture group (*N* = 21) or the sham acupuncture group (*N* = 21) in a 1:1 ratio.

### 3.8. Blinding

Patients, assessors, and data analysts will be blinded. Acupuncture providers will not.

### 3.9. Usual care

Participants will routinely enter into an abstinence procedure, and will be given replacement drugs according to their conditions at initiation in order to control acute withdrawal symptoms such as tremors and irritation (diazepam, for example, at a total dose of 15–100 mg/day, will be administered several times according to the degree of alcohol addiction). According to the conditions, the dose may be tapered every 1–2 days until discontinuation (62). Symptomatic and supportive treatment will also be provided (instead of interventions regarding intestinal flora regulation such as taking probiotics, gavage, etc.).

### 3.10. Intervention

According to traditional acupuncture theory and previous RCTs, the acupuncture study interventions were developed by a consensus of corresponding experts. The interventions will be performed by licensed acupuncturists with over 3 years of clinical experience, who will obtain a total understanding of the treatment based on special training and who will receive a brochure showing the acupuncture manipulation, with detailed information provided before initiation. Subjects who will be randomized to the acupuncture group will receive usual care and JTN treatment for 30 min per session. Meanwhile, those in the control group will receive usual care and sham JTN treatment. From the day after hospital admission for abstinence, acupuncture intervention will be performed once a day for the first 3 days (if the patient is sober) and once every other day thereafter for a total of 2 weeks (8 sessions). This will be followed by a follow-up period lasting until the end of the third month. If the patient presents severe delirium during the last acute withdrawal or has a delirium episode during admission, taking safety into account, the initial acupuncture intervention will not be performed until the delirium recedes and consciousness becomes clear.

#### 3.10.1. Acupoints

**Figure 2:**
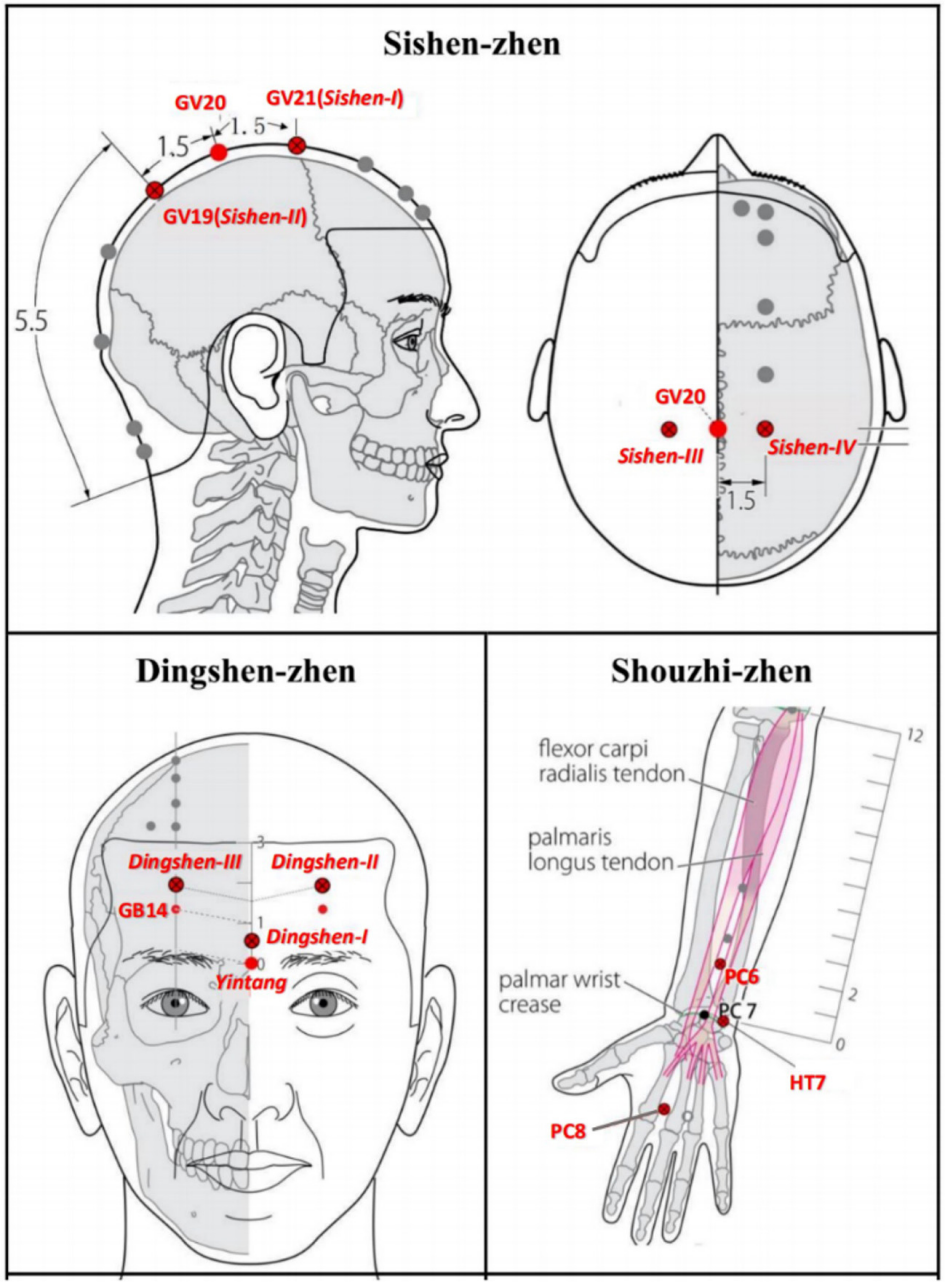
JTN acupoint groups (Sishen-zhen, Dingshen-zhen, and Shouzhi-zhen).

Based on JTN theory, each acupoint group has a special name and contains three or four points. A previous study on acupuncture detoxification Wen et al., (63) showed an effective reduction of opioid craving, and improvement of protracted withdrawal symptoms if three acupoint JTN groups (Sishen-zhen, Dingshen-zhen, and Shouzhi-zhen) were added to methadone replacement therapy (63, 64). Therefore, this set of acupoints will also be chosen in this study (see Figure 2 and Section 6, Supplementary Table S4 in Appendix 1).

#### 3.10.2. Acupuncture device

**Acupuncture needle:** sterile stainless-steel disposable acupuncture needles (Huatuo, Suzhou, China; diameters and lengths of 0.3 mm × 40 mm or 0.25 mm × 25 mm) will be used in the acupuncture group. Blunt acupuncture needles (0.3 mm × 25 mm) will be used in the sham acupuncture group.

**Figure 3:**
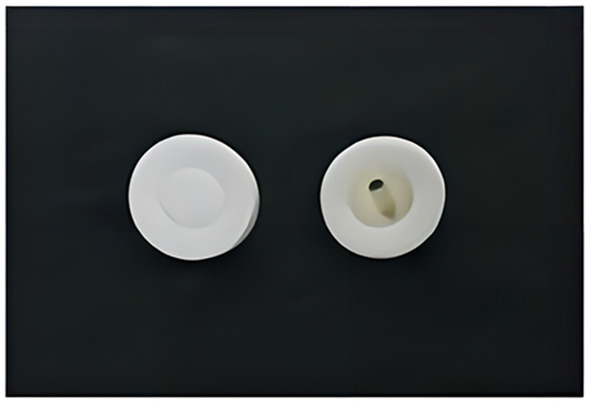
Different types of appliance (left: non-hollow patch for sham acupuncture; right: hollow patch for real acupuncture).

**Placebo device:** a real needle device will be applied in the acupuncture group; a disposable placebo needle device (material: acrylonitrile butadiene styrene plastic, ABS) with a hollow patch on the bottom (material: foam substrate, double-sided, coated with acrylic glue). A sham needle device will be applied in the sham group; a disposable placebo needle device with a mouthless patch on the bottom, made from the same material as the acupuncture group device. The device appearance for both groups is exactly the same. The device has been granted a patent (ZL202121352221.7) by the State Intellectual Property Administration of China (see Figure 3 and Section 6.2 in Appendix 1) (65).

#### 3.10.3. Acupuncture group

**Figure 4:**
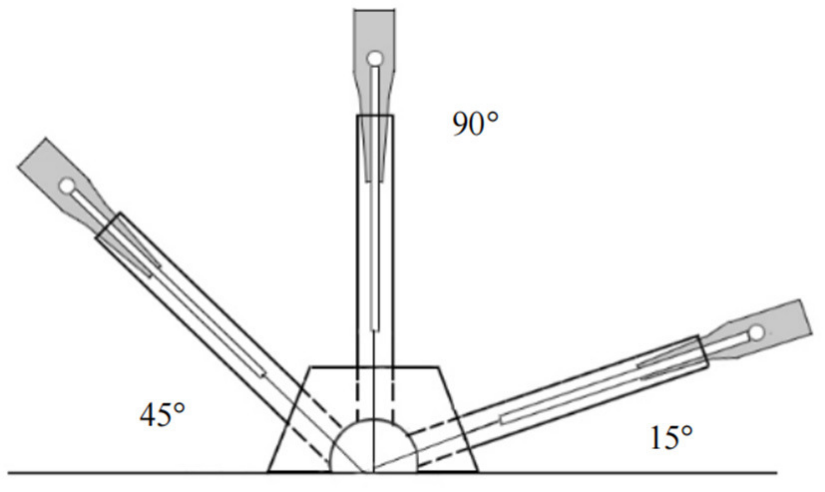
Acupuncture insertion angles.

The corresponding real needle devices will be inserted at Shouzhi-zhen at an angle of 45–90° to the participant's skin, while at Dingshen-zhen and Sishen-zhen, will be inserted at 15° (Figure 4). The needles will be inserted at depths of 5–30 mm. Subsequently, stimulation to achieve the typical acupuncture sensation of “de qi,” characterized by soreness, numbness, and heaviness will be manually performed. During each session, the acupuncture needles will be retained in the skin for 30 min and twirled every 10 min. The intervention will be discontinued if the patient suffers from any adverse events, and the acupuncturists can decide on termination (63).

#### 3.10.4. Sham acupuncture group

Sham acupuncture at the same acupoints will be added to the usual care as a control. Each blunt needle will be inserted into the mouthless patch of the sham needle device, slightly touching the skin, and causing a pinprick-like sensation, without actually penetrating the skin. The needling directions will be the same as those of the acupuncture group. During needle retention, needle handles will be only touched, without any stimulation by acupuncture manipulation, once every 10 min for a total of 3 touches. The setting of the treatment environment, posture, treatment session, frequency, and treatment duration will be equivalent to the acupuncture group; the assessment and the follow-up times for the control group will match those of the experimental group.

### 3.11. Assessment and analyses

The severity of withdrawal symptom assessments and abstinence craving assessments will be conducted every other day, with other indicators assessed at baseline and at the end of the second week of intervention.

## 4. Clinical psychiatric symptom sub-study

### 4.1. Overview of outcome collection

Demographic characteristics will be collected at baseline, and will include age, gender, ethnicity, height, weight, marital status, education level, education duration, occupation, long-term residence, alcohol use (history, common types of drinks, drinking rhythm, frequency, amount, times of treatment for abstinence, etc.), cognitive function, alcohol craving, withdrawal symptoms, accompanying symptoms (mood dysfunction, sleeping disturbance, and fatigue), self-efficacy, and quality of life; replacement drug use will be recorded daily, alcohol craving and the degree of withdrawal symptoms will be recorded every other day for the duration of treatment; alcohol craving, withdrawal symptoms, accompanying symptoms, self-efficacy, and quality of life will be recorded at the end of the last intervention; and drinking self-management and relapse will be recorded at the 3 month follow-up (see Section 7.1, Supplementary Table S5 in Appendix 1). Outcomes are listed in Section 7.2, Supplementary Table S6 in Appendix 1 and their measurements are described in Section 7.3 in Appendix 1, with the measurement tool (scales) index in Section 8 in Appendix 1 and the operative document in Appendix 3. Safety will also be recorded.

### 4.2. Outcomes and measurements

#### 4.2.1. Primary outcome

As an indicator of how quickly alcohol withdrawal symptoms subside, the time required for the symptoms to subside will be assessed according to the **Clinical Institute Alcohol Withdrawal Syndrome Scale** (CIWA-Ar) (66). CIWA-Ar is a 10-point scale for diagnosis, assessment, and guidance for intervention, with the highest score of 67 points possible. According to the ABHGMU model, levels are defined as follows: a total score of 7–9 for mild, 10–18 for moderate, and >18 for severe. The primary outcome is the time required for the CIWA-Ar score to change from ≥ 7 to <7.

#### 4.2.2. Key secondary outcomes

**(1) Change of the alcohol withdrawal symptoms:** the change in CIWA-Ar scores after intervention (ΔCIWA-Ar).

**(2) Change of the withdrawal craving:** visual analog-rating scale (VAS) (67), with the range 0–100 mm used to evaluate the craving derived from alcohol abstinence (abs-VAS) (68). A change of the withdrawal craving is indicated by the change of abs-VAS after intervention (Δabs-VAS).

#### 4.2.3. Secondary outcomes

**(1) Change of the alcohol urge:** the alcohol urge questionnaire (AUQ) is an 8-item, 7-level self-evaluation questionnaire covering three fields of craving (cue-induced craving, expectation for positive effect from drinking, and compulsion) (69). Since VAS is an abstract and unidimensional psychological scale, in order to more comprehensively take into account changes in the patients' positive and compulsive cravings, the change in AUQ (ΔAUQ) on day 14, relative to the baseline, will be used for supplementary measurement of Δabs-VAS.

**(2) Change of cue-induced craving:** VAS, with a 0–100 mm range, will also be used to evaluate the craving derived from alcohol-related cues (cue-VAS) (68, 70, 71). A change of the cue-induced craving is indicated by the change of cue-VAS after the last intervention, relative to the baseline (Δcue-VAS).

**(3) Change of alcohol-related mood dysfunction:** ① depression: the beck depression inventory II (BDI-II) (72) is a 21-question measurement with four-level scoring, from 0 (normality) to 3 (extreme deterioration). A higher score indicates more severe depression: 0–13 = no depression, 14–19 = mild, 20–28 = moderate, and 29–63 = serious. ② Anxiety: the beck anxiety inventory (BAI) (73) is a 21-question measurement with four-level scoring, from 1 (normality) to 4 (extreme deterioration). A total score of 15–25 = mild, 26–35 = moderate, and > 36 = serious. A change of the alcohol-related mood dysfunction is indicated by the change of BDI-II and BAI after the last intervention, relative to the baseline (ΔBDI-II and ΔBAI).

**(4) Improvement of sleep disorder and the sense of fatigue:** ① the Pittsburgh sleep quality index (PSQI) (74) is an 18-item and 7-component measurement of the sleep status, with each component scored according to grade 0–3; the cumulative score is the total PSQI score, with a full score of 21 points possible. The higher the score of each item, the worse the sleep: 0–5, 6–10, 11–15, and 16–21 points indicate good, general, barely acceptable, and poor sleep quality, respectively. ② The mental fatigue scale (MFS) (75) is mainly used to evaluate the mental fatigue of people with neuropsychiatric disorders, and includes 15 items and four options (0, 1, 2, and 3 for no problem, problem existing, with a significant problem, and with a severe problem, respectively) per item for a the total score of 42 (≥ 10.5 is considered as mental fatigue), except for the last item (the change of 24 h fatigue). The higher the score, the more serious the mental fatigue. An improvement of sleep disorder and the sense of fatigue is indicated by the change of PSQI and MFS after the last intervention, relative to the baseline (ΔPSQI and ΔMFS).

**(5) Other secondary outcomes:** other secondary outcomes include changes in the self-efficacy, quality of life, and digestive system dysfunction, the measurement of which are described in Section 7.3 of Appendix 1. The relative scales are presented in Appendix 3.

#### 4.2.4. Follow-up outcomes

**(1) Percentage of heavy drinking days (PHDD), percentage of days abstinent (PDA), drinks per drinking day (DDD), drinks per heavy drinking day (DHDD):** these are indicators of drinking self-management. With monthly timeline followback (TLFB) (76) applied, patients will be asked to review their drinking days for the prior month, and the amount drunk per drinking day, which will be calculated to measure their level of abstinence and their ability to control alcohol intake.

**(2) Relapse or not:** this study will focus on the somatic relapse (re-exposure to alcohol and return to obsession and compulsive desire) (77, 78); relapse will be defined by an AUDIT score ≥ 20 (79) or a Michigan alcohol screening test (MAST) score ≥4 (weighting method) (80) to evaluate whether patients are trapped in relapse, which may require further forced withdrawal treatment during the 3 month follow-up. AUDIT (79) is a questionnaire containing a total of 10 questions and is used to screen for risky and harmful drinking and alcohol dependence. According to the 8-point grading scale, 0–7 points indicates no or mild drinking problems (risk level zone I), 8–15 points indicates moderate drinking problems (zone II), 16–19 points indicates high drinking problems (zone III), and ≥ 20 points may indicate alcohol dependence (zone IV). MAST is a 25-item self-evaluation questionnaire, compiled by referring to the Li version (80). According to the weighting method, a MAST score ≤ 3 can be regarded as having no clinical significance, 4 points indicates possible or suspected alcohol dependence, 5–6 points indicates mild dependence, 7–25 points indicates moderate dependence, 26–39 points indicates serious dependence, and 40–53 points indicates severe dependence. These scales will be used at baseline and at the end of the third follow-up month.

### 4.3. Statistical analysis plan

In this study, the primary outcome and key secondary outcomes will be analyzed using intention to treat (ITT); other outcomes will be analyzed using the PP set. Suspicious results in the analysis process will be discussed using sensitivity analysis. Imputation of missing data will be performed according to the characteristics of the data.

Descriptive statistics will be presented as mean change ± SD (95% confidence intervals) or as a percentage for each group. When normally distributed, and with homogeneous variances, the continuous outcomes will be compared between the groups by independent sample *t*-test, with significance if the *P*-value is < 0.05 (two-sided). Tests will be adjusted if variance is heterogeneous; non-parametric tests will be used for between-group comparisons for abnormal distributions. The Chi-square (χ^2^) test or Fisher's exact test will be used to compare unordered categorical variables between the groups; the rank sum test will be used to compare ordered variables between the groups. Repeated measures analysis of variance or survival time analysis will be used if necessary for outcomes with multiple observation points. For more exploratory analyses, within group comparison may be utilized. SPSS statistical software version 23 (IBM SPSS Statistics, New York, USA) will be used.

## 5. Gut microbiome and fecal metabolomics sub-study

### 5.1. Data preprocessing

**Gut microbiome:** paired-end reads of the raw sequencing data will be preprocessed using cutadapt software to detect and cut off the adapter. After trimmed and low quality sequences filtered, paired-end reads will be denoised, merged, and detected, and the chimera reads will be cut off. Finally, the software will output the representative reads and the ASV abundance table. The representative read of each ASV will be selected using the QIIME2 package. All representative reads will be annotated and blasted against Silva database Version 138 (or Unite) (16s/18s/ITS rDNA) using a q2-feature-classifier with default parameter (81, 82).

**Fecal metabolomics:** a data-independent acquisition (DIA) approach will be applied to simultaneously acquire all fragmented ions of all precursors, thereby increasing the coverage of observable molecules and reducing false negative identification. A series of processing on imported data will be performed, such as peak detection, peak identification, MS2Dec deconvolution (83), characterization, peak alignment, filtering, and missing value imputation.

### 5.2. Data analyses

**Fecal microbiome:** (1) analysis of community structure (differences in the abundance of microorganisms at each taxonomic level): this will mainly involve annotation and abundance summary at each taxonomic level. (2) Biodiversity analysis: ① alpha diversity analysis will be used to learn about the diversity of gut microbiome in each group; ② beta diversity analysis will be used to learn about the diversity of biological environments. (3) Differential microorganism analysis: microbial multivariate statistical analysis will be used to determine and compare significantly different microorganisms between the groups.

**Fecal metabolomics:** (1) multivariate statistical analysis will first use unsupervised principal component analysis (PCA) to observe the overall distributions between samples and the stability of the entire analysis process, then, supervised partial least squares analysis (PLS-DA) and orthogonal partial least squares analysis (OPLS-DA) will be utilized to distinguish overall differences in metabolic profiles between the groups and to determine differential metabolites (84). (2) Univariate analysis will mainly involve interval estimation and statistical hypothesis testing. *T*-test (student's *t*-test) and fold change analysis will be used to compare metabolites between the two groups. (3) Metabolic pathway enrichment analysis of differential metabolites will be based on the KEGG database (85, 86).

The details were provided in Section 9 of Appendix 1.

## 6. Joint analysis sub-study

### 6.1. Omics conjoint analysis

(1) Expression correlation analysis: the Pearson correlation algorithm will be used to calculate the correlation between fecal 16s rRNA and metabolites; (2) pathway mapping: for differential microbiome and metabolites, simultaneous mapping to the pathway database will be performed to obtain common pathway information. (3) Other exploratory analyses will be considered.

### 6.2. Feature screening and predictive model construction

To determine the factors affecting the response to acupuncture, factors such as baseline demographic characteristics, alcohol use, relevant gut microbial and metabolomic indicators obtained using the above steps, and intervention methods (acupuncture/sham acupuncture) will be used as representative feature variables; the outcomes will be used as dependent variables (response variables) to construct corresponding datasets. Feature screening algorithms (based on five rules: missing value, single value, correlation, zero importance, and low importance), combined with the tree model, will be used to integrate and evaluate the critical independent variables and their weights affecting each clinical variable in response to acupuncture.

Considering the small sample size, the transfer learning method will be employed to adapt the predictive model from the source domain to the target domain. First, a general model will be built using the existing public omics data (source domain), and then the model will be fine-tuned using backpropagation on the target domain data in order to adapt the model to the characteristics of the target domain, and, finally, the model will be cross-validated.

The details were provided in Section 10 of Appendix 1.

## 7. Discussion

### 7.1. Protocol overview

The effect of acupuncture on patients with SUDs was well-supported by much previous empirical evidence; however, to the best of our knowledge, no clinical study applying gut microbiome and fecal metabolomics has been performed for acupuncture treatment in the AUD population. This study describes a prospective, randomized, controlled design, utilizing clinical and multi-omics biological information for integrated analysis of microbiome-gut-brain/behavioral mechanisms to explore the gut microbiome and its metabolism mechanisms upon acupuncture adjunctive intervention in patients with AUD.

### 7.2. Comparison with similar previous studies

Although potential positive results have been presented for acupuncture in clinical practice, limitations include acupoint selection is either too simple (87) or not systematic enough (88). As a well-known acupuncture technique, JTN refined acupoints into many concise acupoint groups to target corresponding diseases, regulating the body and spirit together. A study by Wen et al. showed that this three-JTN-group setting imparts the synergistic and detoxifying effects of acupuncture to substance-dependent patients' detoxification intervention (64). Therefore, this study will continue this acupoint setting strategy. Additionally, in contrast to previous studies (89), the sense of “de qi” instead of aversion, will be the target of the needling stimulus manipulation.

Similar to most previous studies, sham acupuncture along with usual care will be applied (51, 89, 90). In contrast, considering the population (familiar with acupuncture) and the specificity of the acupoint effect, a placebo acupuncture device will be used, which has been verified to maintain a suitable blinding effect. This study will carry out rigorous clinical research, conduct an in-depth and systematic analysis of the clinical mechanism of acupuncture for alcohol withdrawal, and apply artificial intelligence algorithms to analyze multi-dimensional data, different from previous studies.

Moreover, the application of combinatorial analysis of the fecal metabolomics and the gut microbiome to obtain multimodal information on the gut microbial community (53) is also scarce in similar studies. This will facilitate understanding of the links between the characteristics of the microbial community structure and the metabolic function, as well as the microbial-host (91).

### 7.3. Implications for clinical practice

Important variables affecting the clinical response of patients with AUD to acupuncture are expected to be found, and their contributions to the response are expected to be shown, which may act as important and non-invasive biomarkers in predictable AUD management in the future. The microbiome-gut-host mechanism of the response of patients with AUD to acupuncture is expected to be discovered in this study, through the fusion of multi-omics and clinical bio-information analyses.

### 7.4. Advantages of this study

A combination of omics techniques and artificial intelligence will be applied to the analysis of the clinical mechanism of acupuncture for alcohol withdrawal, with the efficient use of multi-dimensional clinical data and biological information. Moreover, in this study, the clinical mechanism of acupuncture in patients with AUD will be discussed from the perspective of the relationship between gut microbes and the host.

## 8. Conclusions

A protocol is described above for the integrative analysis of the clinical and multi-omics bioinformatics of acupuncture used as an adjunctive intervention in patients with AUD, which may help to strengthen clinical evidence of the mechanisms related to acupuncture intervention in patients with AUD, through understanding of the regulatory mechanism of acupuncture in the gut microbiota and its metabolism as well as clinical psychopathological manifestations in patients with AUD.

## Ethics statement

The studies involving human participants were reviewed and approved by Institutional Review Board of The Affifiliated Brain Hospital of Guangzhou Medical University. The patients/participants provided their written informed consent to participate in this study.

## Author contributions

CT, LL, and NF proposed the concept for this trial and designed the study. PZ and XLa contributed equally to the conception, design, and manuscript writing. BF, YC, and XW helped search the literature and assisted in the recruitment of patients. BF, YC, and XLi participated in the revision and editing of this manuscript. All authors approved the final version of the manuscript.
