# Supplementary material for: A protocol for the integration of multi-omics bioinformatics: Mechanism of acupuncture as an adjunctive therapy for alcohol use disorder
Source: Front Neurol. 2023 Jan 5;13:977487. doi: 10.3389/fneur.2022.977487 (PMC9849375; doi:10.3389/fneur.2022.977487)
Supplement: Supplementary file 1 [file Data_Sheet_1.docx]

A Protocol for [the Integration of Multi-omics Bioinformatics Mechanism of Acupuncture as an Adjunctive Therapy for Alcohol Use Disorder](http://www.chictr.org.cn/edit.aspx?pid=164634&htm=4)

Supplementary Material summary manual

**Content (totally 10 sections)**

[1 SPIRIT checklist 3](#_Toc16710)

[2 STRICTA checklist 8](#_Toc19087)

[3 Abbreviations 10](#_Toc20499)

[4 Alcohol use disorder in DSM-V 11](#_Toc22043)

[5 Informed consent 12](#_Toc20537)

[6 Acupuncture setting 12](#_Toc3436)

[6.1 Acupoint setting 12](#_Toc8859)

[6.2 Placebo device 13](#_Toc20069)

[7 Clinical outcomes and measurements 14](#_Toc21492)

[7.1 Data recorded 14](#_Toc1966)

[7.2 Clinical outcome setting 17](#_Toc16832)

[7.3 Introductions of measurements 19](#_Toc27775)

[8 Relevant scales 23](#_Toc5971)

[9 Collection of stool samples, data preprocessing, and data-analysis procedure 24](#_Toc13265)

[9.1 Sample collection 24](#_Toc27864)

[9.2 16s rRNA preprocessing 24](#_Toc9712)

[9.2.1 DNA extraction 24](#_Toc24331)

[9.2.2 Library Construction 24](#_Toc5063)

[9.2.3 Bioinformatic analysis 24](#_Toc28128)

[9.2.4 Statistical analysis 25](#_Toc27993)

[9.3 Sample preparation for metabolomics 25](#_Toc8589)

[9.3.1 Data preprocessing 26](#_Toc6474)

[9.3.2 Data analysis 28](#_Toc12410)

[10 Conjoint analysis sub-study 31](#_Toc32099)

[10.1 Omics conjoint analysis 31](#_Toc30253)

[10.2 Feature screening and predictive model construction 31](#_Toc26737)

[10.2.1 Data source and data preprocessing 32](#_Toc18994)

[10.2.2 Deep neural network modeling 35](#_Toc20470)

[10.2.3 Transfer learning 36](#_Toc32643)

[10.2.4 The performance of the model 36](#_Toc10369)

# SPIRIT checklist


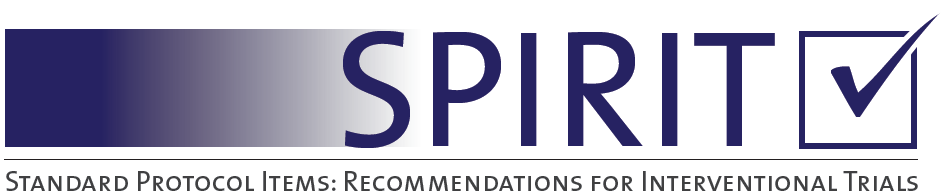


**Supplementary Table 1. SPIRIT 2013 Checklist: Recommended items to address in a clinical trial protocol and related documents***

| **Section/item Item Description**  **Addressed on**  **page number No** | | |
| --- | --- | --- |
| **Administrative information** | | |
| Title | 1 | Descriptive title identifying the study design, population, interventions, and, if applicable, trial acronym P1 |
| Trial registration | 2a | Trial identifier and registry name. If not yet registered, name of intended registery  P2 |
|  | 2b | All items from the World Health Organization Trial Registration Data Set OK |
| Protocol version | 3 | Date and version identifier P11 |
| Funding | 4 | Sources and types of financial, material, and other support P13 |
| Roles and responsibilities | 5a | Names, affiliations, and roles of protocol contributors P1,12 |
|  | 5b | Name and contact information for the trial sponsor P1 |
|  | 5c | Role of study sponsor and funders, if any, in study design; collection, management, analysis, and interpretation of data; writing of the report; and the decision to submit the report for publication, including whether they will have ultimate authority over any of these activities P13 |
|  | 5d | Composition, roles, and responsibilities of the coordinating center, steering committee, endpoint adjudication committee, data management team, and other individuals or groups overseeing the trial, if applicable (see Item 21a for data monitoring committee) P13 |
| **Introduction** | | |
| Background and rationale | 6a | Description of research question and justification for undertaking the trial, including summary of relevant studies (published and unpublished) examining benefits and harms for each intervention P2-4 |
|  | 6b | Explanation for choice of comparators P2-4 |
| Objectives | 7 | Specific objectives or hypotheses P2-4 |
| Trial design | 8 | Description of trial design including type of trial (eg, parallel group, crossover, factorial, single group), allocation ratio, and framework (eg, superiority, equivalence, noninferiority, exploratory) P4 |
| **Methods: Participants, interventions, and outcomes** | | |
| Study setting | 9 | Description of study settings (eg, community clinic, academic hospital) and list of countries where data will be collected. Reference to where list of study sites can be obtained P4 |
| Eligibility criteria | 10 | Inclusion and exclusion criteria for participants. If applicable, eligibility criteria for study centers and individuals who will perform the interventions (eg, surgeons, psychotherapists) P4-5 |
| Interventions | 11a | Interventions for each group with sufficient detail to allow replication, including how and when they will be administered P5-6 |
|  | 11b | Criteria for discontinuing or modifying allocated interventions for a given trial participant (eg, drug dose change in response to harms, participant request, or improving/worsening disease) P6 |
|  | 11c | Strategies to improve adherence to intervention protocols, and any procedures for monitoring adherence (eg, drug tablet return, laboratory tests) P5-6 |
|  | 11d | Relevant concomitant care and interventions that are permitted or prohibited during the trial P5 |
| Outcomes | 12 | Primary, secondary, and other outcomes, including the specific measurement variable (eg, systolic blood pressure), analysis metric (eg, change from baseline, final value, time to event), method of aggregation (eg, median, proportion), and time point for each outcome. Explanation of the clinical relevance of chosen efficacy and harm outcomes is strongly recommended P8-9 |
| Participant timeline | 13 | Time schedule of enrolment, interventions (including any run-ins and washouts), assessments, and visits for participants. A schematic diagram is highly recommended P4 (see Figure 1) |
| Sample size | 14 | Estimated number of participants needed to achieve study objectives and how it was determined, including clinical and statistical assumptions supporting any sample size calculations P5 |
| Recruitment | 15 | Strategies for achieving adequate participant enrolment to reach target sample size P5 |
| **Methods: Assignment of interventions (for controlled trials)** | | |
| Allocation: |  |  |
| Sequence generation | 16a | Method of generating the allocation sequence (eg, computer-generated random numbers), and list of any factors for stratification. To reduce predictability of a random sequence, details of any planned restriction (eg, blocking) should be provided in a separate document that is unavailable to those who enrol participants or assign interventions P6 |
| Allocation concealment mechanism | 16b | Mechanism of implementing the allocation sequence (eg, central telephone; sequentially numbered, opaque, sealed envelopes), describing any steps to conceal the sequence until interventions are assigned P6 |
| Implementation | 16c | Who will generate the allocation sequence, who will enrol participants, and who will assign participants to interventions P6 |
| Blinding (masking) | 17a | Who will be blinded after assignment to interventions (eg, trial participants, care providers, outcome assessors, data analysts), and how P6 |
|  | 17b | If blinded, circumstances under which unblinding is permissible, and procedure for revealing a participant’s allocated intervention during the trial P6 |
| **Methods: Data collection, management, and analysis** | | |
| Data collection methods | 18a | Plans for assessment and collection of outcome, baseline, and other trial data, including any related processes to promote data quality (eg, duplicate measurements, training of assessors) and a description of study instruments (eg, questionnaires, laboratory tests) along with their reliability and validity, if known. Reference to where data collection forms can be found, if not in the protocol  P7-9 |
|  | 18b | Plans to promote participant retention and complete follow-up, including list of any outcome data to be collected for participants who discontinue or deviate from intervention protocols Not applicable |
| Data management | 19 | Plans for data entry, coding, security, and storage, including any related processes to promote data quality (eg, double data entry; range checks for data values). Reference to where details of data management procedures can be found, if not in the protocol  as Hao Wen 2020 described(doi:  [10.1186/s13063-020-04930-x](https://doi.org/10.1186/s13063-020-04930-x)) |
| Statistical methods | 20a | Statistical methods for analysing primary and secondary outcomes. Reference to where other details of the statistical analysis plan can be found, if not in the protocol P9-11 |
|  | 20b | Methods for any additional analyses (eg, subgroup and adjusted analyses) P9-11 |
|  | 20c | Definition of analysis population relating to protocol non-adherence (eg, as randomized analysis), and any statistical methods to handle missing data (eg, multiple imputation) P9 |
| **Methods: Monitoring** | | |
| Data monitoring | 21a | Composition of data monitoring committee (DMC); summary of its role and reporting structure; statement of whether it is independent from the sponsor and competing interests; and reference to where further details about its charter can be found, if not in the protocol. Alternatively, an explanation of why a DMC is not needed  as Hao Wen 2020 described(doi:  [10.1186/s13063-020-04930-x](https://doi.org/10.1186/s13063-020-04930-x)) |
|  | 21b | Description of any interim analyses and stopping guidelines, including who will have access to these interim results and make the final decision to terminate the trial  as Hao Wen 2020 described(doi:  [10.1186/s13063-020-04930-x](https://doi.org/10.1186/s13063-020-04930-x)) |
| Harms | 22 | Plans for collecting, assessing, reporting, and managing solicited and spontaneously reported adverse events and other unintended effects of trial interventions or trial conduct P8 |
| Auditing | 23 | Frequency and procedures for auditing trial conduct, if any, and whether the process will be independent from investigators and the sponsor  as Hao Wen 2020 described(doi:  [10.1186/s13063-020-04930-x](https://doi.org/10.1186/s13063-020-04930-x)) |
| **Ethics and dissemination** | | |
| Research ethics approval | 24 | Plans for seeking research ethics committee/institutional review board (REC/IRB) approval  P12 |
| Protocol amendments | 25 | Plans for communicating important protocol modifications (eg, changes to eligibility criteria, outcomes, analyses) to relevant parties (eg, investigators, REC/IRBs, trial participants, trial registries, journals, regulators) P13 |
| Consent or assent | 26a | Who will obtain informed consent or assent from potential trial participants or authorised surrogates, and how (see Item 32) P5 |
|  | 26b | Additional consent provisions for collection and use of participant data and biological specimens in ancillary studies, if applicable  Appendix 2 |
| Confidentiality | 27 | How personal information about potential and enrolled participants will be collected, shared, and maintained in order to protect confidentiality before, during, and after the trial  as Hao Wen 2020 described(doi:  [10.1186/s13063-020-04930-x](https://doi.org/10.1186/s13063-020-04930-x)) |
| Declaration of interests | 28 | Financial and other competing interests for principal investigators for the overall trial and each study site P12 |
| Access to data | 29 | Statement of who will have access to the final trial dataset, and disclosure of contractual agreements that limit such access for investigators  as Hao Wen 2020 described(doi:  [10.1186/s13063-020-04930-x](https://doi.org/10.1186/s13063-020-04930-x)) |
| Ancillary and post-trial care | 30 | Provisions, if any, for ancillary and post-trial care, and for compensation to those who suffer harm from trial participation  as Hao Wen 2020 described(doi:  [10.1186/s13063-020-04930-x](https://doi.org/10.1186/s13063-020-04930-x)) |
| Dissemination policy | 31a | Plans for investigators and sponsor to communicate trial results to participants, healthcare professionals, the public, and other relevant groups (eg, via publication, reporting in results databases, or other data sharing arrangements), including any publication restrictions  as Hao Wen 2020 described(doi:  [10.1186/s13063-020-04930-x](https://doi.org/10.1186/s13063-020-04930-x)) |
|  | 31b | Authorship eligibility guidelines and any intended use of professional writers （Trial participants, no planned use of professional writers） |
|  | 31c | Plans, if any, for granting public access to the full protocol, participant-level dataset, and statistical code  as Hao Wen 2020 described(doi:  [10.1186/s13063-020-04930-x](https://doi.org/10.1186/s13063-020-04930-x)) |
| **Appendices** | | |
| Informed consent materials | 32 | Model consent form and other related documentation given to participants and authorised surrogates Appendix 2 |
| Biological specimens | 33 | Plans for collection, laboratory evaluation, and storage of biological specimens for genetic or molecular analysis in the current trial and for future use in ancillary studies, if applicable Appendix 1 Section 9 |

*It is strongly recommended that this checklist be read in conjunction with the SPIRIT 2013 Explanation & Elaboration for important clarification on the items. Amendments to the protocol should be tracked and dated. The SPIRIT checklist is copyrighted by the SPIRIT Group under the Creative Commons “[Attribution-NonCommercial-NoDerivs 3.0 U](http://www.creativecommons.org/licenses/by-nc-nd/3.0/)[nported](http://www.creativecommons.org/licenses/by-nc-nd/3.0/)” license.

# STRICTA checklist

**Supplementary Table 2. Standards for Reporting Interventions in Clinical Trials of Acupuncture(** **STRICTA )**

| **Section/topic** | **Item number** | | | **Checklist item** | **Report/**  **Not** |
| --- | --- | --- | --- | --- | --- |
| 1.Acupuncture rationale | | | | | |
|  | | 1a | Style of acupuncture (eg, Traditional Chinese Medicine, Japanese, Korean, Western medical, Five Element, ear acupuncture, etc) . | |  |
|  |  | 1b | Reasoning for treatment provided, based on historical context, literature sources and/or consensus methods, with references where appropriate . | |  |
|  |  | 1c | Extent to which treatment was varied . | | |
| 2.Details of needling | |  |  | |  |
|  | | 2a | Number of needle insertions per subject per session (mean and range where relevant) | |  |
|  |  | 2b | Names (or location if no standard name) of points used (uni-/bilateral) . | |  |
|  | | 2c | Depth of insertion, based on a specified unit of measurement or on a particular tissue level . | |  |
|  |  | 2d | Responses sought (eg, *de qi* or muscle twitch response) . | |  |
|  | | 2e | Needle stimulation (eg, manual or electrical) . | |  |
|  |  | 2f | Needle retention time . | |  |
|  | | 2g | Needle type (diameter, length and manufacturer or material) . | |  |
| 3.Treatment regimen | |  |  | |  |
|  | | 3a | Number of treatment sessions . | |  |
|  |  | 3b | Frequency and duration of treatment sessions . | |  |
| 4.Other components of treatment | |  |  | |  |
|  | | 4a | Details of other interventions administered to the acupuncture group (eg, moxibustion, cupping, herbs, exercises, lifestyle advice) . | |  |
|  |  | 4b | Setting and context of treatment, including instructions to practitioners, and information and explanations to patients . | |  |
| 5.Practitioner  background | |  |  | |  |
|  | | 5 | Description of participating acupuncturists (qualification or professional affiliation, years in acupuncture practice, other relevant experience) . | |  |
| 6.Control or comparator  interventions | |  |  | |  |
|  | | 6a | Rationale for the control or comparator in the context of the research question, with sources that justify the choice(s). | |  |
|  | | 6b | Precise description of the control or comparator. If sham acupuncture or any other type of acupuncture-like control is used, provide details as for items 1-3 above. | |  |

This checklist should be read in conjunction with the explanations of the Standards for Reporting Interventions in Clinical Trials of Acupuncture items.

# Abbreviations

AUDIT: Alcohol Use Disorders Identification Test

AUQ: Alcohol Urge Questionnaire

MAST: Michigan Alcohol Screening Test

ABHGMU: Affiliated Brain Hospital of Guangzhou Medical University

AIDS: Acquired Immunodeficiency Syndrome

ANOVA: analysis of variance

AUD: Alcohol use disorder

AUDIT: Alcohol Use Disorder Identification Test

AUQ: Alcohol Urge Questionnaire

BAI: Beck Anxiety Inventory

BPRS: Brief Psychiatric Rating Scale

BDI-II: Beck Depression Inventory II

CIWA-Ar: Clinical Institute Alcohol Withdrawal Syndrome Scale

DIA: data independent acquisition

DDD: Drinks per drinking day

DHDD: Drinks per heavy drinking day

DSM-V: Diagnostic and Statistical Manual of Mental Disorders Fifth Edition

FDR: false discovery rate

GSES: General Self-Efficacy Scale

GSRS: Gastrointestinal symptom rating scale

GZUCM: Guangzhou University of Chinese Medicine

JTN: Jin’s three-needle acupuncture

MAST: Michigan Alcohol Screening Test

MFS: Mental fatigue scale

MMSE: Minimum Mental State Examination

OTUs: Operational Taxonomic Units

PD: Phylogenetic Diversity

PDA: Percentage of days abstinent

PHDD: Percentage of heavy drinking days

PLS-DA: partial least Squares-discriminant analysis

PSQI: Pittsburgh sleep quality index

QOL-DA: Quality of life scale for patients with drug addiction/dependence

RCT: Randomized controlled trial

SPIRIT: Standard Protocol Items: Recommendations for Interventional Trials

STRICTA: Standards for Reporting Interventions in Controlled Trials of Acupuncture

SUDs: substance use disorders

TLFB: Timeline Followback

VAS: Visual Analogue-rating scale

VIP: variable importance in projection

# Alcohol use disorder in DSM-V[1]

**Supplementary Table 3. DSM-V diagnostic criteria for alcohol use disorder**

| **The disease diagnosed** | **Items** |
| --- | --- |
| **Alcohol use disorder** (A problematic pattern of alcohol use leading to clinically significant impairment or distress, as manifested by at least two of the following, occurring within a 12-month period) | 1. Alcohol is often taken in larger amounts or over a longer period than was intended. |
|  | 2. There is a persistent desire or unsuccessful efforts to cut down or control alcohol use. |
|  | 3. A great deal of time is spent in activities necessary to obtain alcohol, use alcohol, or recover from its effects. |
|  | 4. Craving, or a strong desire or urge to use alcohol. |
|  | 5. Recurrent alcohol use resulting in a failure to fulfill major role obligations at work, school, or home. |
|  | 6. Continued alcohol use despite having persistent or recurrent social or interpersonal problems caused or exacerbated by the effects of alcohol. |
|  | 7. Important social, occupational, or recreational activities are given up or reduced because of alcohol use. |
|  | 8. Recurrent alcohol use in situations in which it is physically hazardous. |
|  | 9. Alcohol use is continued despite knowledge of having a persistent or recurrent physical or psychological problem that is likely to have been caused or exacerbated by alcohol. |
|  | 10. Tolerance, as defined by either of the following:  a A need for markedly increased amounts of alcohol to achieve intoxication or desired effect.  b A markedly diminished effect with continued use of the same amount of alcohol. |
|  | 1. Withdrawal, as manifested by either of the following:   a The characteristic withdrawal syndrome for alcohol (refer to Criteria A and B of the criteria set for alcohol withdrawal, pp. 499–500).  b Alcohol (or a closely related substance, such as a benzodiazepine) is taken to  relieve or avoid withdrawal symptoms. |

If participants is diagnosed with AUD by DSM-V but the alcohol use problem is less than 1 year, as long as any of the following condition is met, it will also be eligible: (1) The ALcohol Use Disorders Identification Test (AUDIT) ≥ 20 indicates there is ongoing or highly suspected alcohol dependence and necessary to enter an alcohol intervention program, or (2) Michigan Alcohol Screening Test (MAST) (weighting method) ≥ 4 indicates that the person may experience or is experiencing alcohol dependence.

**Reference**

1. Association AP. *Diagnostic and Statistical Manual of Mental Disorders*. Arlington, VA: American Psychiatric Publishing; (2013).
2. Babor, T. F., Higgins-Biddle, J. C., Saunders, J. B., Monteiro, M. G. *The alcohol use disorders identification test*. Geneva: World Health Organization; (2001).
3. Zhang M. *Handbook of psychiatric assessment scales 2nd Edition*. Changsha, Hunan, China: Hunan Science and Technology Press; (1998).

# Informed consent

The informed consent is in Appendix 2.

# Acupuncture setting

**6.1 Acupoint setting**

**Supplementary Table 4. Location and insertion depth of acupoints**

| **Acupoints** | **Description** | **Insersion depth（mm）** | **Insersion**  **Angle** |
| --- | --- | --- | --- |
| 1. **Sishen-zhen** | **Including Sishen-I, II, III, IV** | 20-25 | 15–30° |
| GV19: Qianding (*Sishen-I*) | On the head, 3.5 B-cun superior to the anterior hairline, on the anterior median line. | - | 15–30° |
| GV21: Houding (*Sishen-II*) | On the head, 5.5 B-cun superior to the posterior hairline, on the posterior median line. | - | 15–30° |
| GV20: Baihui | On the head, 5 B-cun superior to the anterior hairline, on the anterior median line. | - | - |
| *Sishen-III* | On the head, 1.5 cun left lateral to the anterior median line and at the same level as GV20. | - | 15–30° |
| *Sishen-IV* | On the head, 1.5 cun right lateral to the anterior median line and at the same level as GV20. | - | 15–30° |
| 1. **Dingshen-zhen** | **Including Dingshen-I, II, III** | 20-25 | 15–30° |
| EX-NH3: Yintang | On the head, between the right medial end of the eyebrow and the left one. | - | - |
| ***Dingshen-I*** | On the head, directly 0.5cun superior to EX-HN3. | - | 15–30° |
| GB14: Yangbai | On the head, 1B-cun superior to the eyebrow, directly superior to the center of the pupil. | - | - |
| ***Dingshen-II*** | On the head, directly 0.5cun superior to left GB14. | - | 15–30° |
| ***Dingshen-III*** | On the head, directly 0.5cun superior to right GB14. | - | 15–30° |
| 1. **Shouzhi-zhen** | **Including PC6, HT7, PC8** | - | 45–90° |
| HT7: Shenmen | On the anteromedial aspect of the wrist, radial to the flexor carpi ulnaris tendon, on the palmar wrist crease. | 20-25 | 90° |
| PC6: Neiguan | On the anterior aspect of the forearm, between the tendons of the palmaris longus and the flexor carpi radialis, 2B-cun proximal to the palmar wrist crease. | 5-10 | 90° |
| PC8: Laogong | On the palm, in the depression between the second and third metacarpal bones, proximal to the metacarpophalangeal joints. | 5-10 | 45°toward LI 4 (Hegu) |

**Note:** The prescribed acupoints come from the WHO Standard Acupuncture Point Locations in the Western Pacific Region

**6.2 Placebo device**

To match acupuncture clinical study practically, our team will apply a placebo acupuncture appliance, which can adjust different needling angles. The appliance has already granted the patent by China national intellectual property administration (No. ZL202121352221.7). The placebo acupuncture appliance contains a pedestal and a tube. The pedestal, made by resin material, is opaque so that it can isolate patients’ eyesight,and they cannot visually decide whether the needle is inserted into skin or not. There is a sticky patch in the base of the pedestal to ensure the pedestal can stick to the skin. The tube is transparent so that it is convenient for doctors to insert the needle through the tube. In addition, This device has two types of pedestals. One is hollowed in the base, the other is not hollowed . For the sake of real acupuncture, we use the hollowed appliance, and the needle can insert into the skin directly and hygienically. For the sake of placebo acupuncture, we use the nonhollowed appliance and the blunt needle. The sticky base can clamp the non-invasive needle so that the needle will not drop whenever patients move. For different angles, there are three holes in the pedestal, one on the top and two on the sides. If putting the tube in the top hole, then the needle can hold a straight angle while putting the tube in the side hole, the needle is beveled to the skin that can match the need of needling in scalp.

# Clinical outcomes and measurements

## Data recorded

**Supplementary Table 5. Clinical Data collection**

| **Aspect** | **Content** | **Measurements** | **Time point of record** |
| --- | --- | --- | --- |
| Demographic characteristics | Age  Gender  Ethnicity (Minzu)  Height  Weight  Marital status  Education level  Occupation  Family history  Underlying health conditions | Demographic forms | Baseline |
| Cognitive and memory function | - | MMSE | Baseline |
| Vital signs | body temperature  Pulse  Respiration  Blood pressure  Heart rate. | Physical examination | Baseline |
| Alcohol use | Drinking years  Common types of drinks  Rhythm  Times of treatment for abstinence  Primary drinking motivation  Voluntary admission for treatment  Self-reported abstinence goal | TLFB | Baseline |
|  | Frequency  Amount | TLFB | Baseline & follow up |
|  | problematic drinking behavior and risk | AUDIT | Baseline & follow up |
|  | Alcohol dependence | MAST | Baseline & follow up |
| Alcohol-withdrawal symptoms | Alcohol withdrwal craving | abs-VAS | Baseline & treatment duration |
|  |  | AUQ | Baseline & endpoint |
|  | Alcohol withdrawal syndrome | CIWA-Ar | Baseline & treatment duration |
|  |  | Time required for withdrawal symptoms to subside | Endpoint (based on the daily records) |
|  | Replacement drug use | - | Endpoint (based on the daily records) |
|  | Gastrointestinal withdrawal reactions | GSRS | Baseline & endpoint |
| Mood dysfunction | Depression | BDI-II | Baseline & endpoint |
|  | Anxiety | BAI | Baseline & endpoint |
| Sleep quality and sense of fatigue | Sleep quality | PSQI | Baseline & endpoint |
|  | Sense of fatigue | MFS | Baseline & endpoint |
|  | - |  |  |
| Self-efficacy of Alcohol abstinence | - | GSES | Baseline & endpoint |
| Quality of life | - | QOL-DA | Baseline & endpoint |
| Cue-realated alcohol craving | - | cue-VAS | Baseline & endpoint |
| Relapse | DDD  DHDD  PDA  PHDD | TLFB | Follow up (based on the drinking frequency and amount records) |
|  | Relapse or not | AUDIT/MAST | Follow up |

## Clinical outcome setting

**Supplementary Table 6. Clinical outcome setting**

| **Aspect** | **Content** | **Measurements** |
| --- | --- | --- |
| Primary outcome | Time required for alcohol withdrawal symptoms to subside | CIWA-Ar |
| Key secondary outcomes | Change of the alcohol withdrawal symptoms | CIWA-Ar |
|  | Change of the withdrawal craving | abs-VAS |
| Secondary outcomes | Change of the alcohol urge | AUQ |
|  | Change of the cue-induced craving | cue-VAS |
|  | Change of the depression | BDI-II |
|  | Change of the anxiety | BAI |
|  | Improvement of the sleep disorder | PSQI |
|  | Improvement of the sense of fatigue | MFS |
|  | Change of the self-efficacy | GSES |
|  | Change of the digestive system dysfunction | GSRS |
|  | Change of the quality of life | QOL-DA |
| Follow-up outcomes | DDD  DHDD  PDA  PHDD | TLFB |
|  | Relapse rate | AUDIT/MAST |

**Note:**

AUDIT: Alcohol Use Disorder Identification Test

AUQ: Alcohol Urge Questionnaire

BAI: Beck Anxiety Inventory

BDI-II: Beck Depression Inventory II

CIWA-Ar: Clinical Institute Alcohol Withdrawal Syndrome Scale

DDD: Drinks per drinking day

DHDD: Drinks per heavy drinking day

GSES: General Self-Efficacy Scale

GSRS: Gastrointestinal symptom rating scale

MAST: Michigan Alcohol Screening Test

MFS: Mental fatigue scale

MMSE: Minimum Mental State Examination

PDA: Percentage of days abstinent

PHDD: Percentage of heavy drinking days

PSQI: Pittsburgh sleep quality index

QOL-DA: Quality of life scale for patients with drug addiction/dependence

TLFB: Timeline Followback

VAS: Visual Analogue-rating scale

## Introductions of measurements

**(1) Score of problematic drinking behavior and risk leveling - AUDIT[1]:** The scale screens risky and harmful drinking and alcohol dependence, with a total of 10 questions covering alcohol consumption, frequency, alcohol dependence items, forgetting, etc. The first 8 questions are 5-level scoring and the last 2 are 3-level scoring. According to the 8-point grading boundary, 0-7 points indicate no or mild drinking problems (risk level Zone I), 8-15 points indicate moderate drinking problems (Zone II), 16-19 points indicate high drinking problems (Zone III), and ≥20 points may be alcohol dependence (Zone IV). Patients in Zone III will be treated as those in Zone IV if they are still suspected of alcohol dependence after a brief consultation and continuous monitoring. This scale will be used at baseline and the end of the third follow-up month.

**(2) Questionnair of Alcohol dependence - Michigan Alcohol Screening Test (MAST):** a 25-item self-evaluation questionnaire referring to Lingjiang Li version[2], except the Item 0 is for introduction, the others include physical and psychological dependence, and the effects of drinking on body and mind, occupation, and social function, etc. The total score indicates the severity of alcohol problems: 0 no drinking, 1-2 low drinking, 3-5 light drinking, 6-13 moderate drinking, 14-20 heavy drinking, 21-24 severe drinking. According to the weighting method, MAST ≤ 3 points can be regarded as having no clinical significance; 4 points are possible or suspected alcohol dependent objects; 5-6 points indicate mild dependence; 7-25 points are moderate; 26-39 points are serious; 40-53 points are severe.

**(3) Assessment of Alcohol craving:** Distinction between the two definitions of cue-induced craving and withdrawal psychological craving proposed was by Drummond, who believes that the latter belongs to withdrawal symptoms (mainly aversive response), and the former prompts the possibility and psychological expectations for patients to obtain the substance (mainly reward response), different with the latter, and perhaps more close to the relapse [3]. Therefore, the **Alcohol Urge Questionnaire (AUQ),** an 8-item 7-Level self-evaluation questionnaire covering 3 fields of craving (craving, expectation for positive effect from drinking, and compulsion)[4], and **Visual Analogue-rating scale (VAS)[5]** with 0-100 mm ranging will be used to evaluate the two craving types derived respectively from withdrawal (abs-VAS) and cue-induced (cue-VAS).

First, the withdrawal craving will be evaluated by VAS, and the urge to drink during the withdrawal period by AUQ; then subjects will be instructed to imagine and describe the drinking details for 3 min of the drinking scenario after the longest abstinence period ever experienced, and then the craving during the imagining process will be evaluated using VAS[6-7]. Cue induction will be assessed only at baseline and after the last treatment, for avoiding the interference caused by multiple cue exposures.

**(4) Assessment of Alcohol withdrawal symptoms:** ①**Clinical Institute Alcohol Withdrawal Syndrome Scale (CIWA-Ar)[8]:** CIWA-Ar is a 10-dimension scale for diagnosis, assessment, and guidance for intervention, with the highest score of 67 points. According to the ABHGMU model, the levels are defined as: a total score of 7-9 for mild, 10-18 for moderate, >18 for severe. ②**Time required for the complete regression of the withdrawal symptoms (CIWA<7):** as an indirect indicator, the speed of withdrawal symptom regression will be assessed according to the CIWA-Ar results. ③**abs-VAS and AUQ** (see 7.3 (3)): abs-VAS and AUQ wll be supplemented because CIWA-Ar does not cover the withdrawal craving. ④**Replacement drug use:** It is an Indirectly reflection of the relief of the acute alcohol withdrawal symptoms. Patients' daily replacement drug dosage will be monitored and recorded.⑤Gastrointestinal withdrawal reactions(see 7.3 (10)): Gastrointestinal symptom rating scale (GSRS) will be supplemented for withdrawal symptoms of digestive system because only partial common withdrawal gastrointestinal symptoms covered by CIWA-Ar.

**(5) Assessment of Alcohol-related mood dysfunction:** ①Depression - **Beck Depression Inventory II (BDI-II)[9]:** It is a 21-question measurement with 4-level scoring from 0 (normality) - 3 (extreme deterioration). The higher score means the more severe: 0-13= no depression, 14-19= mild, 20-28= moderate, 29-63= serious. ②Anxiety - **Beck Anxiety Inventory (BAI)[10]:** It is a 21-question measurement with 4-level scoring from 1 (normality) - 4 (extreme deterioration): the total score 15-25=mild, 26-35=moderate, >36=serious.

**(6) Assessment of Sleep quality and sense of fatigue:** ①**Pittsburgh sleep quality index (PSQI)[11]:** It is an 18-item and 7-component measurement for the sleep status during abstinence, including sleep quality, sleep latency, sleep duration, sleep efficiency, sleep disturbances, use of sleeping medications and daytime dysfunction. Each component is scored according to grades 0-3, of which the cumulative score is the total PSQI score, with a full score of 21 points. The higher the score of each item, the worse the sleep: 0-5 points, 6-10 points, 11-15 points, 16-21 points respectively for good, general, barely acceptable, and poor sleep quality. The Chinese PSQI scale will be used in this study and the assessment interval will be set at 2 weeks. ②**Mental fatigue scale (MFS)[12]:** it is mainly used to evaluate the mental fatigue of people with neuropsychiatric disorders. The scale combines the changes of emotion, cognition, fatigue, sleep, and 24h fatigue severity, with 15 items. Except for the last item (the change of 24h fatigue), each item has four options (0, 1, 2, 3 respectively for no problem, problem existing, with a significant problem, and with a severe problem). If the choice is in the middle of the two options, they will be rated as 0.5, 1.5, and 2.5. And except for the last item, the total score is 42. The higher the score, the more serious the mental fatigue is. A score≥10.5 is considered mental fatigue.

**(7)Impairment of cognitive and memory function - Minimum Mental State Examination (MMSE)[13]:** It has 30 questions, scoring 1 point for each correct answer, and 0 for incorrect or unknown. The dividing value between normal and abnormal is related to education level: ≤17 points for illiterate (uneducated), ≤20 points for primary school (≤6 years of education), and ≤24 points for middle school or above (years of education> 6 years). Below the cut-off value indicates a cognitive function defect, while the above indicates normal cognitive function.

**(8) Self-efficacy of Alcohol withdrawal - General Self-Efficacy Scale (GSES)[14]:** Self-efficacy refers to the patient's control or dominance of their behaviors, and reflects the belief that patients themself can take appropriate actions facing the environmental challenges. The stronger self-efficacy will provide more probability for patients to cooperate with withdrawal treatment more spontaneously and actively, and treat the encountered pressure in the wiithdrawal process more confidently, so it is closely related to the withdrawal prognosis. This 10-item self-evaluation scale applies the Likert 4-point system, with each item scoring "completely incorrect", "a bit correct", "mostly correct" and "completely correct" recorded 1-4 points respectively.

**(9) Quality of life - quality of life scale for patients with drug addiction/dependence (QOL-DA,** Chonghua Wan, Jiqian Fang, et al.**)[15]:** Considering the characteristics of the target population and their quality of life assessment, they are often more concerned with the effect of medical interventions. This measurement is divided into four dimensions: physical function (9 items, 45 points), psychological function (9 items, 45 points), symptoms/side effects (11 items, 55 points), and social function (11 items, 55 points), belonging to 41 items, each item 5 levels of 1-5 scores, with positive items 33-40 and the rest reverse scoring. Each dimension item has a sub-total score, and the sum is 200 points. Higher scores indicate better quality of life/health status.

**(10) Digestive system dysfunction -** The Gastrointestinal symptom rating scale (GSRS)[16] is a 15-item specific scale used to assess gastrointestinal symptoms over the past week, including 5 gastrointestinal symptoms reflux, abdominal pain, dyspepsia, diarrhea, and constipation. The GSRS takes a 7-point Likert rating scale ranging from "asymptomatic" to "very severe"; the higher the score, the more severe the symptoms.

**(11) Follow-up measurements:** ①**Indicators of drinking self-management:** Monthly **Timeline Followback (TLFB)[17]** applied, patients will be asked, to review the drinking days of the last month (involving the first time of alcohol exposure again), the drink times and amount per drinking day, which will be calculated as Drinks per drinking day (DDD), Drinks per heavy drinking day (DHDD), Percentage of days abstinent (PDA), Percentage of heavy drinking days (PHDD) to measure their level of abstinence and ability to control alcohol intake. ②**Relapse or not:** it means returning to compulsive alcohol seeking and drinking after a period of constant sobriety, and reproducing the addictive characteristics of unhealthy behaviors and negative consequences. It is difficult to define a relapse by one intake, and generally, relapse progresses through three stages: emotional relapse - mental relapse - somatic relapse, involving obvious warning signs like talking about a lack of drinking, traveling with drinking partners, anxious or depressed, becoming more isolated, missing sessions or treatment appointments, and unhealthy eating[18-19]. These three stages have still not been clearly defined due to the mixing and inconsistency of the characteristics. Given that, this study will focus on the somatic relapse (re-exposing to alcohol and return to obsession and compulsive desire), and the relapse will be defined by AUDIT≥20 or MAST (weighting method) ≥4, to evaluate whether patients trap in a relapse which may require another forced withdrawal treatment during the three-month follow-up. ③Three-month relapse rate: Relapse rate = number of relapse cases / total cases * 100%.

**Reference**

1. Babor, T. F., Higgins-Biddle, J. C., Saunders, J. B., Monteiro, M. G. *The alcohol use disorders identification test*. Geneva: World Health Organization; (2001).

2. Zhang M. *Handbook of psychiatric assessment scales 2nd Edition*. Changsha, Hunan, China: Hunan Science and Technology Press; (1998).

3. Bohn MJ, Krahn DD, Staehler BA. Development and Initial Validation of a Measure of Drinking Urges in Abstinent Alcoholics. *Alcohol Clin Exp Res* (1995) 19:600–606. doi: https://doi.org/10.1111/j.1530-0277.1995.tb01554.x

4. Wewers ME, Lowe NK. A critical review of visual analogue scales in the measurement of clinical phenomena. *Res Nurs Health* (1990) 13:227–236. doi: https://doi.org/10.1002/nur.4770130405

5. Drummond DC. Theories of drug craving, ancient and modern. *Addiction* (2001) 96:33–46. doi: https://doi.org/10.1046/j.1360-0443.2001.961333.x

6. Vorspan F, Fortias M, Zerdazi E, Karsinti E, Bloch V, Lépine J-P et al. Self-reported cue-induced physical symptoms of craving as an indicator of cocaine dependence. *Am J Addict* (2015) 24:740–743. doi: https://doi.org/10.1111/ajad.12303

7. Dong P, Xin X, Ni Z, Tao R, Sun H. Latent phenomenon of psychological craving of alcohol dependent patients after withdrawal in real environment. *Chinese Journal of drug dependence* (2017) 26(2): 104-108.

8. SULLIVAN JT, SYKORA K, SCHNEIDERMAN J, NARANJO CA, SELLERS EM. Assessment of Alcohol Withdrawal: the revised clinical institute withdrawal assessment for alcohol scale (CIWA-Ar). *Br J Addict* (1989) 84:1353–1357. doi: https://doi.org/10.1111/j.1360-0443.1989.tb00737.x

9. Beck A T, Ward C H, Mendelson M, et al. An inventory for measuring depression. *Archives of general psychiatry* (1961) 4(6): 561-571.

10. Beck AT, Epstein N, Brown G, Steer RA. An inventory for measuring clinical anxiety: Psychometric properties. *J Consult Clin Psychol* (1988) 56:893–897. doi: 10.1037/0022-006X.56.6.893

11. Buysse DJ, Reynolds CF, Monk TH, Berman SR, Kupfer DJ. The Pittsburgh sleep quality index: A new instrument for psychiatric practice and research. *Psychiatry Res* (1989) 28:193–213. doi: https://doi.org/10.1016/0165-1781(89)90047-4

12. Johansson B, Starmark A, Berglund P, Rödholm M, Rönnbäck L. A self-assessment questionnaire for mental fatigue and related symptoms after neurological disorders and injuries. *Brain Inj* (2010) 24:2–12. doi: 10.3109/02699050903452961

13. Mowla A, Zandi T. Mini-mental status examination: a screening instrument for cognitive and mood disorders of elderly. *Alzheimer Disease & Associated Disorders* (2006) 20(2): 124.

14. Luszczynska A, Scholz U, Schwarzer R. The general self-efficacy scale: multicultural validation studies. *The Journal of psychology* (2005) 139(5): 439-457.

15. Wan C, Fang J, Chen L, He L, Gao Y. Development and evaluation of quality of life scale for drug addicts. *Chinese behavioral medicine science* (1997) (03):11-13.

16. Dimenäs E, Glise H, Hallerbäck B, et al. Quality of life in patients with upper gastrointestinal symptoms: an improved evaluation of treatment regimens?. *Scandinavian journal of gastroenterology* (1993) 28(8): 681-687.

17. Sobell LC, Sobell MB. “Timeline Follow-Back BT  - Measuring Alcohol Consumption: Psychosocial and Biochemical Methods.,” In: Litten RZ, Allen JP, editors. Totowa, NJ: Humana Press (1992). p. 41–72 doi: 10.1007/978-1-4612-0357-5_3

18. American Society of Addiction Medicine. *Terminology related to addiction, treatment, and recovery*; (2013).

19. Chris Elkins. Alcohol Relapse. MADrugRehab.com. (2020)[2022-02-20]. Available from: <https://www.drugrehab.com/addiction/alcohol/relapse/#sources.>

# Relevant scales

The relevant scales are in Appendix 3.

# Collection of stool samples, data preprocessing, and data-analysis procedure

## Sample collection

Stool samples will be collected in sterile tubes, stored in a −80°C freezer immediately, and transported to OE Biotech Co., Ltd.

## 16s rRNA preprocessing

9.2.1 DNA extraction

Total genomic DNA will be extracted using DNA Extraction Kit (MagPure Soil DNA LQ Kit, Magen, Guangzhou, China) following the manufacturer’s instructions. Concentration of DNA will be verified with NanoDrop and agarose gel. The genome DNA will be used as template for PCR amplification with the barcoded primers and Tks Gflex DNA Polymerase (Takara). For bacterial diversity analysis, V3-V4 (or V4-V5) variable regions of 16S rRNA genes will be amplified with universal primers 343 F and 798 R (or 515F and 907R for V4-V5 regions). For eukaryota diversity analysis, variable regions of 18S rRNA genes will be amplified with universal primers 817F and 1196R. For fungal diversity analysis, ITS I variable regions will amplified with universal primers ITS1F and ITS2[1-6].

9.2.2 Library Construction

Amplicon quality will be visualized using gel electrophoresis, purified with AMPure XP beads (Agencourt), and amplified for another round of PCR. After purified with the AMPure XP beads again, the final amplicon will be quantified using Qubit dsDNA assay kit. Equal amounts of purified amplicon will be pooled for subsequent sequencing.

9.2.3 Bioinformatic analysis

The 16S/18S/ITS amplicon sequencing and analysis will be conducted by OE biotech Co., Ltd. (Shanghai, China).

Raw sequencing data will be in FASTQ format. Paired-end reads will then be preprocessed using cutadapt software to detect and cut off the adapter. After trimming, paired-end reads will be filtering low quality sequences, denoised , merged and detect and cut off the chimera reads using DADA2 with the default parameters of QIIME2 (2020.11). At last, the software will output the representative reads and the ASV abundance table. The representative read of each ASV will be selected using QIIME2 package. All representative reads will be annotated and blasted against Silva database Version 138 (or Unite) (16s/18s/ITS rDNA) using q2-feature-classifier with the default parameter[7-8].

### 9.2.4 Statistical analysis

The analyses will include assessing the biological richness and diversity of the samples within the group, community similarities, and differences between different biological environments (different subgroups), and seeking differential microbes:

Gut microbial abundance, ei. the number of taxonomic levels of intestinal microorganisms, will be measured by the number of tags of Amplicon Sequencing Variants (ASVs).

Biodiversity of gut microbes will be measured by the alpha diversity index. Common alpha diversity indexes: (1) The **Shannon-Weiner diversity index**[9] is used to describe the disorder and uncertainty of individuals of different species, involving two factors (richness (number of species) and evenness of individual distribution in various species). the higher Shannon index indicates the higher diversity. (2) **Simpson's diversity index**[10] is used to evaluate the probability of different species obtained by random sampling, reflecting the status of the dominant species in the community. (3) **Phylogenetic Diversity (PD) whole tree**[11] is also a diversity index calculated based on the phylogenetic tree (PT). The PT is constructed from the representative sequences of OTUs in each sample, which is the sum of the branch lengths of all representative sequences in a sample. The higher PD whole tree indicates the higher biodiversity. (4) **Chao1 index**[12] is the number of OTUs contained in the sample community estimated by the Chao1 algorithm. The larger Chao1 value indicates the greater total number of species. (5) **Good's Coverage**[13] is the sequencing depth index, which represents the ratio of all non-singletons in the total sample. In theory, with the increase of sequencing depth, if the singleton no longer appears, it indicates that sequencing has covered all species in the sample. In the case of an equal amount of sequencing data, the larger Good's Coverage value indicates the lower biodiversity of the sample.

Beta diversity will describe the similarities and differences in gut biological environments between groups.

ANOVA and Kruskal Wallis algorithms will be used to calculate the species with significant differences at different taxonomic levels between groups, and then the top 10 with different abundances will be selected for relative abundance analysis to obtain the result of abundance comparison of dominantly differential species within and between groups. LEfSe will be used to deeply analyze the contribution of different species to the difference.

In addition, Databases Kyoto Encyclopedia of Genes and Genomes (KEGG) and Clusters of Orthologous Genes (COGs) will be used for community function prediction to understand the functional differences of gut microbes between different groups or between pre- and post-intervention.

## Sample preparation for metabolomics

We will try to analyze fecal metabonomics by double platform (Gas Chromatography-Mass Spectrometry and Liquid Chromatography–Mass Spectrometry, GC-MS and LC-MS). Samples will be extracted and added with internal standard for GC-MS and LC-MS analyses. Here we mainly showed the protocol of GC-MS.

### 9.3.1 Data preprocessing

GS condition: DB-5MS capillary column (30 m × 0.25mm × 0.25 μm, Agilent J&W Scientific, Folsom, CA, USA) will be applied with the high-purity helium (purity not less than 99.999%) as the carrier gas, the flow rate 1.0 mL/min, the temperature of the injection port 260 °C, the splitless injection 1 μL, and the solvent delay 6.2 min. Programmable temperature: the initial temperature of the column oven will be 60 °C for 0.5 min; it will be heated up successively to 125 °C and 210 °C at 8 °C/min, to 270 °C at 15 °C/min, and to 305 °C at 20 °C/min, and then be hold for 5 min[14].

MS condition: Electron bombardment ion source (EI), ion source temperature 230 °C, quadrupole temperature 150 °C, electron energy 70 eV, scan mode: full scan (SCAN), mass scan range: m/z 50-500[15].

A data-independent acquisition (DIA) approach will be applied to simultaneously acquire all fragment ions of all precursors, thereby increasing the coverage of observable molecules and reducing false negative identification. MS-DIAL is based on the qualitative and quantitative analysis of small molecular mass deconvolution in DIA mode[16]. The raw data (D format) of GC/MS will be converted into the basic file with abf format for analysis by Analysis Base File Converter software for fast data retrieval, and then it will be imported into MS-DIAL software for preprocessing to efficiently find precursor ion peaks by analyzing two consecutive data axes, with MS2Dec algorithm used for compound characterization through extracting "model peaks" in the chromatogram, removing background noise, passing retention time, refining molecular mass, and similarity-matching to public databases. MS-DIAL will also implement additional features required for off-target metabolomics, such as peak alignment, filtering, and missing value imputation. MS-DIAL performs a series of processing on imported data, such as peak detection, peak identification, MS2Dec deconvolution, characterization, peak alignment, filtering, and missing value imputation. Qualitative analysis of metabolites will be based on the LUG database and finally the original data matrix will be exported, which will include: sample information, the name of each substance peak, retention time, mass-to-charge ratio, and mass spectral response intensity (peak area).

GC-MS database: The LUG database (Untarget database of GC-MS from Lumingbio) independently developed by Lumingbio, which contains 2543 metabolites that can be detected by GC-MS, and is constantly updated, will be applied for GC-MS characterization. Cytoplasm-nucleus ratios range from 85 to 650, covering lipids, amino acids, fatty acids, amines, ethanols, Carbohydrates, aminoglycosides, glycitols, sugar acids, organophosphates, hydroxy acids, [Aromatic compounds](http://dict.cn/Aromatic compounds), purines, sterols, and other substances. The information of metabolites in the LUG database contains HMDB, KEGG, CAS identification numbers, substance classifications, etc., which is convenient for subsequent metabolite function research. For headspace sampling GC-MS experiments investigating volatiles and similar substances, the NIST database (https://webbook.nist.gov/chemistry/ ) will be used for qualitative analysis of the substances. The NIST database is generally considered an EI-MS database, of which the latest version also includes ESI MS/MS mass spectra of small molecule compounds, including metabolite chemical standards, lipids, and bioactive peptides[16].

Qualitative and quantitative results: The internal standard will be used for data quality control, and the internal standard peaks in the raw data matrix and any known false positive peaks (including noise, column bleed, and derivatizing reagent peaks) will be removed, with missing values replaced with zeros. In each sample, all peak signal intensities (peak areas) will be segmented normalized according to the internal standard with RSD (ALL) < 0.3 after screening (ie, according to the internal standard with RSD < 0.3 in all samples after screening and the internal standard response intensity at different retention times, the internal standard normalization of metabolites by retention time segments will be performed, and the signal intensity of each peak will be converted into the relative internal standard intensity in this time segment in the spectrum). After data normalization, redundancy, and merge peaks data will be removed to obtain a data matrix as follows.

**Supplementary Table 7. Major Qualitative result parameters**

| **Parameters** | **Explanation** |
| --- | --- |
| Alignment ID | Metabolite number |
| Average Rt (min) | Average retention time |
| Average RI | Average retention index |
| Quant mass | Molecular mass |
| Metabolites | Metabolite name |
| Total score | Total similarity score |
| HMDB | HMDB library ID |
| METLIN | METLIN library ID |
| Lipidmaps | Lipidmaps library ID |
| pubChem | pubChem library ID |
| kegg | kegg library ID |
| CAS | CAS ID |
| inchikey | Inchikey ID |
| Super Class | Main class |
| Class | Classification |
| Sub Class | Sub-classification |

The matrix contains all the information extracted from the original data that can be used for analysis, and subsequent analysis will be based on this.

### 9.3.2 Data analysis

Multivariate statistical analysis will first use unsupervised principal component analysis (PCA) to observe the overall distribution between samples and the stability of the entire analysis process, and then use supervised partial least squares analysis (PLS-DA) and orthogonal Partial least squares analysis (OPLS-DA) to distinguish overall differences in metabolic profiles between groups and to find differential metabolites[17].

Univariate analysis will mainly focus on the description and statistical inference of univariates, the former using a simplest form to reflect the basic information contained in mountains of sample information and describe the concentration or dispersion trend, and the later inferring the population situation from the sample, mainly involving interval estimation and statistical hypothesis testing. *t* test (Student’s *t* test) and fold change analysis are commonly used to compare metabolites between two groups.

**Supplementary Table 8. Parameters of differential metabolite screening**

| **Parameters** | **Explanation** |
| --- | --- |
| VIP | Variable important in projection; the VIP value from the OPLS-DA model; the larger the VIP, the greater the contribution of the variable to the grouping. |
| *P*-value | The *T*-test results, evaluating whether the variables are significantly different between the two groups; *P*<0.05 means significant. |
| adj. *P*-value | False discovery rate (FDR), used to control the proportion of false positive results in variance analysis, calculated using the *Benjamini-Hochberg* method. |
| FC | The ratio of the average expression of metabolites in two groups of samples |
| Log2(FC) | The average expression ratio of metabolites in the two groups of samples; positive value means up-regulation, while negative value means down-regulation. |
| Average (#) | #The average expression of metabolites in the group |

Metabolic pathway enrichment analysis: The construction of the KEGG (https://www.kegg.jp/) database aims to understand the functions and interactions of genes, proteins, and metabolites in biological systems (such as cells, tissues, etc.). Information on metabolic pathways, human diseases, and drug development related to metabolites can be queried. The metabolites and metabolic pathways in this database involve two broad categories: eukaryotes (animals, plants, fungi, and protists) and prokaryotes (bacteria, archaea). Pathway enrichment analysis of differential metabolites is helpful to understand the mechanism of metabolic pathway changes in differential samples. Metabolic pathway enrichment analysis of differential metabolites will be based on the KEGG database.

**Supplementary Table 9. Parameters of differential metabolite pathway enrichment results**

| **Parameters** | **Explanation** |
| --- | --- |
| ID Annotation | The ID number of the metabolic pathway; the first 3 or 4 letters such as ssc, hsa, ath, etc. indicate the species |
| Annotation | Metabolic pathway name |
| in set | The number of differential metabolites involved in certain pathway |
| set | The number of differential metabolites involved in all pathways |
| in background | Total number of metabolites belonging to model species in this pathway |
| RichFactor | Enrichment factor, in set divided by in background |
| *P*-value | Hypergeometric test *P*-value for this metabolic pathway |
| –lg*(P*-value) | The logarithm of the *P*-value to the base 10, the smaller the *P*-value, the larger the –lg(*P*-value) value |
| FDR correction | FRD (False Discovery Rate) corrected *P*-value |
| Matching IDs | Differential metabolite ID numbers involved in this metabolic pathway |
| URL | The KEGG URL of the metabolic pathway |

**Reference**

1. Nossa Carlos W, Oberdorf, William E, Yang Liying et al. Design of 16S rRNA gene primers for 454 pyrosequencing of the human foregut microbiome. *World J. Gastroenterol.* (2010) 16: 4135-44.

2. Wu Liyou, Wen Chongqing, Qin Yujia et al. Phasing amplicon sequencing on Illumina Miseq for robust environmental microbial community analysis. *BMC Microbiol.* (2015) 15: 125.

3. Xiong Jinbo, Liu Yongqin, Lin Xiangui et al. Geographic distance and pH drive bacterial distribution in alkaline lake sediments across Tibetan Plateau. *Environ. Microbiol.* (2012) 14: 2457-66.

4. Rousk Johannes, Bååth Erland, Brookes Philip C et al. Soil bacterial and fungal communities across a pH gradient in an arable soil. *ISME J.* (2010) 4: 1340–1351.

5. Mukherjee Pranab K, Chandra Jyotsna, Retuerto Mauricio et al. Oral mycobiome analysis of HIV-infected patients: identification of Pichia as an antagonist of opportunistic fungi. *PLoS Pathog.* (2014) 10: e1003996.

6. Pires Ana CC, Cleary Daniel FR, Almeida Adelaide et al. Denaturing gradient gel electrophoresis and barcoded pyrosequencing reveal unprecedented archaeal diversity in mangrove sediment and rhizosphere samples. *Appl. Environ. Microbiol.* (2012) 78: 5520-8.

7. Callahan B J , Mcmurdie P J , Rosen M J , et al. DADA2: High-resolution sample inference from Illumina amplicon data. *Nature Methods* 2016.

8. Reproducible, interactive, scalable and extensible microbiome data science using QIIME 2. *Nature Biotechnology* 37(8):852-857.

9. Shannon, C.E. & Weaver, W. *The mathematical theory of communication*. Urbana: The University of Illinois Press; (1949). 117pp.

10. Simpson EH. Measurement of Diversity. *Nature* (1949) 163: 688.

11. Faith DP, Baker AM. Phylogenetic diversity (PD) and biodiversity conservation: some bioinformatics challenges. *Evolutionary Bioinformatics Online* (2005) 2(2): 121-128.

12. Chao A. Non-parametric estimation of the classes in a population. *Scandinavian Journal of Statistics* (1984) 11(4): 265-270.

13. Esty WW. The Efficiency of Good's Nonparametric Coverage Estimator. *Annals of Statistics* (1986) 14(3): 1257-1260.

14. Nicholson J. et al., “Metabonomics”: understanding the metabolic responses of living systems to pathophysiological stimuli via multivariate statistical analysis of biological NMR spectroscopic data. *Xenobiotica.* (1999) 29(11):1181-1189.

15. Kind T. et al. FiehnLib: mass spectral and retention index libraries for metabolomics based on quadrupole and time-of-flight gas chromatography/mass spectrometry.*Anal. Chem.* (2009) 81(24): 10038-10048.

16. Tsugawa H. et al., MS-DIAL: data-independent MS/MS deconvolution for comprehensive metabolome analysis. *Nature Methods*. (2015) 12(6):523-526.

17. Trygg J. et al., Orthogonal projections to latent structures (O-PLS). *J. Chemometr*. (2002) 16(3):119-128.

# Conjoint analysis sub-study

## Omics conjoint analysis

**①Expression correlation analysis:** According to the relative content data between stool 16s rRNA and metabolites, the Pearson correlation algorithm will be used to calculate the correlation between stool 16s rRNA and metabolites. Draw a correlation matrix for the TOP 20 results of the correlation analysis, and differential genera and differential metabolites before and after intervention; use the Pearson correlation algorithm to calculate the correlation between microbial abundance and metabolites response intensity data. Correlation.

**②Pathway mapping:** For differential genera and differential metabolites, simultaneously map to the KEGG pathway database to obtain their common pathway information.

## Feature screening and predictive model construction

To find out the factors affecting the response to acupuncture, factors such as baseline demographic characteristics, alcohol use, and relevant gut microbial and metabolomic indicators obtained in the above steps, and intervention methods (acupuncture/sham acupuncture) will be used as representative feature variables; the outcomes will be used as dependent variables (response variables) to construct datasets correspondingly, respectively. Feature screening algorithms combined with the tree model will be tried to integrate and evaluate the critical independent variables and their weights affecting each clinical variable responding to acupuncture.

This study selects the following five rules to perform feature screening on the features contained in each task data.

①Missing value: It is a common situation that there are missing values in the data set. Generally, in the process of data preprocessing, the missing values will be properly handled. Generally speaking, there are deletion and imputation method to deal with missing values, including mean imputation, interpolation imputation, hot deck imputation, etc. In this study, we will try first simply delete features with a missing value ratio higher than 90%.

②Single value: When the values of a feature vector are all the same value, it is called that the vector has a single value. Obviously, a single value has no reference in the problem of classifying labels, so this part of the variable will be deleted before training the prediction model.

③ Correlation: Correlation is a coefficient that measures the degree of correlation between two feature vectors. Variables with high correlation coefficients may cause collinearity problems, thereby reducing the model accuracy of the classifier. The model will randomly remove a variable with a correlation coefficient value higher than 0.7 in the study.

④Zero importance: First of all, Importance measures the role of feature variables in predicting categorical variables. the Gradient Boosting Tree (GBM) algorithm will be applied with the feature vector x in each task dataset as the input data and the task prediction y as the categorical variable. The output Wi is the importance score of each feature. When the score is zero, it is defined as zero importance, that is, the model judges that the variable has no effect on the classification and prediction results.

⑤ Low importance: Low importance is still measure using the output result of the above gradient boosting tree. First, the importance scores will be sorted from high to low, and add them repeatedly to get the cumulative score curve. Variables with cumulative importance over 0.90 will be defined as low importance. These variables have insignificant contribution in predicting tasks, and produce information redundancy. Therefore, the features marked as low importance will be deleted.

Considering the small sample size, the **transfer learning** method will be tried to adapt the predictive model from the source domain to the target domain:

**10.2.1 Data source and data preprocessing**

The microbiome and metabolome data used in this study will be downloaded from the GEO or published work.

These data will be set as a source domain for deep neural network modeling. As for the target domain, we collect clinical factors (x) from case report forms of recruited participants. Multi-omics data is also included.

For the data, the expression features will be selected based on the features in the target domain data. We will further remove samples with more than 20% missing values. Samples missing clinical endpoint data will also be filtered out. The data matrix will be standardized such that each feature has a zero mean and unit standard deviation. To verify whether there are significant differences between the two groups of data. The ANOVA *F* value for each Multi-omics data will be calculated for the training samples to select Multi-omics data as the input features for machine learning. The feature mask, ANOVA *F* value, and p values will be calculated using the SelectKBest function (with the f_classif score function and k = the amount of omics data) of the python sklearn package[1].

**Supplementary Table 10. Characteristic variable table**

| **Feature variables** | **Independent variables x** | **dependent variables y*** |
| --- | --- | --- |
| Time taken for withdrawal symptoms to fade | x | y |
| Change in depression (BDI-II) | x | y |
| Change in anxiety (BAI) | x | y |
| Change in sleep disturbance (PSQI) | x | y |
| Change in fatigue (MFS) | x | y |
| Change of digestive system symptoms (GSRS) | x | y |
| Change in drinking urge (AUQ) | x | y |
| Post-cue-related craving (cue-VAS) | x | y |
| Change in self-efficacy (GSES) | x | y |
| Change in quality of life (QOL-DA) | x | y |
| Relapse |  | y |
| Age | x |  |
| Nationality (Minzu) | x |  |
| Body mass index (BMI) | x |  |
| Marital status | x |  |
| Education | x |  |
| Job occupation | x |  |
| Working status | x |  |
| Underlying health conditions | x |  |
| Family history | x |  |
| Drinking years | x |  |
| Number of times received systemic AUD intervention (ie. hospitalization for AUD treatment) | x |  |
| Baseline percentage of drinking days (PDD) | x |  |
| Baseline percentage of heavy drinking days (PHDD) | x |  |
| Baseline drinks per drinking day (DDD) | x |  |
| Baseline drinks per heavy drinking day (DHDD) | x |  |
| Drinking rhythm | x |  |
| Type of alcohol drinks | x |  |
| Primary motivation for drinking | x |  |
| Voluntary admission for abstinence | x |  |
| Self-reported alcohol abstinence goals | x |  |
| Total replacement drug dosage | x |  |
| Baseline [AUD identification test](https://www.hepatitis.va.gov/alcohol/treatment/audit-c.asp) (AUDIT) | x |  |
| Baseline severity of alcohol dependence (MAST) | x |  |
| Baseline severity of AWS（CIWA-Ar） | x |  |
| Baseline depression (BDI-II) | x |  |
| Baseline anxiety (BAI) | x |  |
| Baseline sleep disturbance (PSQI) | x |  |
| Baseline fatigue (MFS) | x |  |
| Baseline digestive symptoms (GSRS) | x |  |
| Baseline withdrawal craving (abs-VAS) | x |  |
| Baseline cue-related craving (cue-VAS) | x |  |
| Baseline self-efficacy (GSES) | x |  |
| Baseline quality of life (QOL-DA) | x |  |
| Baseline drinking compulsion (AUQ) | x |  |
| Total dosage of alternative medicines | x |  |
| Receive acupuncture or not | x |  |
| Post-gut microbial abundance | x | y |
| Post-gut biodiversity | x | y |
| Post-differential microbials | x | y |
| Post-differential metabolites | x | y |
| Post-metabolic pathways enriched | x | y |
| Baseline gut microbial abundance | x |  |
| Baseline gut biodiversity | x |  |
| Baseline differential microbials | x |  |
| Baseline differential metabolites | x |  |
| Baseline metabolic pathways enriched | x |  |

**Note:**

MAST: Michigan Alcohol Screening Test

VAS: Visual analog scale

[BDI: Beck Depression Inventory](https://en.wikipedia.org/wiki/Beck_Depression_Inventory)

[BAI: Beck Anxiety Inventory](https://www.jolietcenter.com/storage/app/media/beck-anxiety-inventory.pdf)

MMSE: Mini–Mental State Examination

GSES: General Self-Efficacy Scale

QOL-DA: Quality of Life for Drug Addicts

AUD: Alcohol use disorder

AWS: Alcohol withdrawal symptoms

CIWA-Ar: [Clinical Institute Withdrawal Assessment](https://umem.org/files/uploads/1104212257_CIWA-Ar.pdf) of Alcohol Scale, revised

GSRS: Gastrointestinal symptom rating scale

* When continuous indicators are used as the dependent variable y, some of them will be converted to categorical indicators of acupuncture response. For example, using Δ% = (pre-treatment score - post-treatment score) / pre-treatment score × 100%, the changes of the above indicators after the intervention can be discretized as action parameters, into clinical cure (Δ% ≥ 75%), significant effect (50% ≤ Δ% < 75%), effective (25% ≤ Δ% < 50%), and ineffective (Δ% < 25%).

**10.2.2 Deep neural network modeling**

We used the Theano python packages (http://deeplearning.net/ software/theano/) and Lasagne (https://lasagne. readthedocs.io/en/latest/) to train the DNN.

We used a pyramid architecture[2] with 6 layers: the number of nodes in the input layer is the feature number of omics data, 4 hidden layers including a fully connected layer with 256 nodes followed by a dropout layer, a fully connected layer with 128 nodes followed by a dropout layer, and a logistic regression output layer.

To fit a DNN model, we used the stochastic gradient descent method with a learning rate of 0.001 (lr = 0.001) to find the weights that minimized a loss function consisting of a cross-entropy and L2 regularization term:

$$l\left( W \right)=-\sum_{i = 1}^{n} (y_{i}\log\left( \hat{y}_{i} \right)+\left( 1-y_{i} \right)\log(1-\hat{y}_{i}))+\lambda_{2}\left| \left| W \right| \right|_{2}$$

where $y_{i}$ is the observed label of patient i, $\hat{y}_{i}$ is the predicted label for patient i, and W represents the weights in the DNN.

Traditional activation functions such as the sigmoid and hyperbolic tangent functions have a gradient vanishing problem in training a deep-learning model, which may lead to gradient decreasing quickly and training error propagating to forward layers. Here, we will use the ReLU function f(x) = max (0, x), which is widely used in deep learning to avoid the gradient vanish problem. For each dropout layer, we will set the dropout probability p = 0.5 to randomly omit half of the weights during the training to reduce the collinearity between feature detectors. To speed up the computation, we will split the data into multiple mini-batches during training, and use a batch size of 20 (batch_size = 20) for the source domain if there are relatively large numbers of cases available for training. We will set the maximum number of iterations at 100 (max_iter = 100) and apply the Nesterov momemtum[3] method (with momentum = 0.9 for each DNN model) to avoid premature stopping. The two regularization terms λ2 will be set at 0.001.

**10.2.3 Transfer learning**

For transfer learning, we will set the gut microbiome and fecal metabolome data as the source domain and our study data as the target domain.

We will first pre-train a DNN model using source domain data, without loss of generality, the pretraining process is to minimize the loss L over the source-domain data, resulting in the model parameter θ:

$$\begin{matrix} \mathrm{argmin} \\ \theta\end{matrix}=\sum_{(x,y)\in D_{S}} L_{S}(x,P(y|x),P_{\theta}(y|x))$$

which has the same architecture as described in the previous section. The training parameters will be set as lr = 0.01, batch_size = 20, p = 0.5, max_iter = 100, and momentum = 0.9. The pre-trained model is considered a starting point in the target domain. After the initial training, the DNN model will be then fine-tuned using backpropagation on the target domain data:

$$\begin{matrix} \mathrm{argmin} \\ \theta' \end{matrix}=\sum_{(x,y)\in D_{t}} L_{T}(x,P(y|x),P_{\theta}(y|x))$$

where $\theta'$ will be the final model’s parameter. In the fine-tuning, the learning rate will be set at 0.002 and the batch size will be set at 5 as the model has been partially fitted and the target dataset will be small.

**10.2.4 The performance of the model**

To see how well the models are performing, we will conduct 10-fold cross-validation on the source domain and at each iteration, 80% of data will be used for training, 10% for validation, and 10 for testing. The results of the cross-validation folds will be finally pooled to calculate the overall metrics, including accuracy, F1-score.

**Reference**

1. Pedregosa F, Varoquaux G, Gramfort A, Michel V, Thirion B, Grisel O et al. Scikit-learn: machine learning in Python. J. *Mach. Learn. Res.* (2011) 12, 2825–2830.

2. Phung, S. L. & Bouzerdoum, A. A pyramidal neural network for visual pattern recognition. *IEEE Trans. Neural Netw.* (2007) 18, 329–343.

3. Sutskever, I., Martens, J., Dahl, G. & Hinton, G. On the importance of initialization and momentum in deep learning. *In International Conference on Machine Learning* (2013) 1139–1147.
