# Supplementary material for: A protocol for the integration of multi-omics bioinformatics: Mechanism of acupuncture as an adjunctive therapy for alcohol use disorder
Source: Front Neurol. 2023 Jan 5;13:977487. doi: 10.3389/fneur.2022.977487 (PMC9849375; doi:10.3389/fneur.2022.977487)
Supplement: Supplementary file 2 [file Data_Sheet_2.docx]

**A Protocol for [the Integration of Multi-omics Bioinformatics Mechanism of Acupuncture as an Adjunctive Therapy for Alcohol Use Disorder](http://www.chictr.org.cn/edit.aspx?pid=164634&htm=4)**

Informed Consent

### Informed consent for clinical research (intervention study)

**Project Title:** [Mechanism of Acupuncture as an Adjunctive Therapy for Alcohol Use Disorder](http://www.chictr.org.cn/edit.aspx?pid=164634&htm=4) Based on Multi-modal Bioinformatics.

**Sponsor:** Xiaochang Lan

**Clinical trial institution:** Affiliated Brain Hospital of Guangzhou Medical University (Guangzhou Hui'ai Hospital)

**Version or Date:** V1.0 February 22, 2022

**Principal researcher:** Xiaochang Lan

**Personal reading material**

**Dear Patient, please read this article carefully and your questions and discussions with your family, relatives, friends, or us are welcome.**

You are invited to participate in this clinical study, whose objective is to explore the mechanism of acupuncture as an adjunctive intervention for alcohol use disorder (AUD), provide it with a safe and effective TCM treatment method, and deeply understand its clinical efficacy mechanism.

Whether or not to participate in this study depends entirely on you, so please read this material carefully before you make a decision. It can help you gain a comprehensive understanding of the purpose, methodology, and process of the study, and the benefits and inconveniences of participating in the study. The information provided to you in this informed consent form can help you decide whether to participate in it, and please ask the researcher responsible for the project if you have any questions, or discuss it with your family, relatives, and friends to ensure that you fully understand the content. Your participation is voluntary, and if you agree to participate in it, please sign the statement of informed consent.

1. **What is the study? [Study background and purpose]**

The title of this study is *The* *[Mechanism of Acupuncture as an Adjunctive Therapy for Alcohol Use Disorder](http://www.chictr.org.cn/edit.aspx?pid=164634&htm=4) Based on Multi-modal Bioinformatics*. The objective of the study is to explore the mechanism of acupuncture adjunctive intervention for AUD, provide it with a safe and effective TCM treatment method, and understand how it works more deeply. This study has been reviewed by Guangzhou Hui'ai Hospital and has been considered following the *Declaration of Helsinki* and in line with medical ethics.

Research Background:

AUD, as one of the most prevalent chronic recurrent substance use disorders (SUDs), has a widespread negative impact on global public health. However, the current shortage of treatment options for the disease, its potential contraindications, and the high rates of relapse suggest that alternative treatments or optimized treatment strategies need to be considered to promote cost-effective policies and interventions for AUD intervention. Since the 1970s, acupuncture for the treatment of SUDs has achieved great fruit, and Jin three needles (JTN) from the 1980s began to be applied to the clinics, one of whose characteristics is mind regulation, with the remarkable effect on mental diseases such as anxiety, depression, sleep dysfunction. One of our previous studies showed that JTN therapy used for opioid addiction reduced the withdrawal symptoms and the consumption of methadone replacement. Growing evidence highlights the role of gut-brain mechanisms in the development of AUD, with previous studies suggesting that acupuncture may work by modulating gut-brain mechanisms. Finding clinical biomarkers of acupuncture as an adjunctive intervention for AUD and exploring its mechanisms are of great significance for the decision-making of evidence-based personalized withdrawal strategies in the future.

Participating center: Guangzhou Huiai Hospital

Total number of cases expected: 42

Study timeline: April 2022 - March 2023

1. **Is participation in this study voluntary?**

Participation is voluntary, which means you have the right to decide whether or not to participate in this study, and no reason is required for the rejection, without any discrimination, retaliation, and destroyment of your medical relationship or interest at all, and you can continue to receive treatment from your doctor.

1. **Who is not suitable to participate in it**

You are not suitable to participate in this study if you have any of the following:

(1)Accompanied by serious neurological or psychiatric diseases caused by diseases other than alcohol dependence (including brain tissue damage caused by traumatic brain injury);

(2)Use of other psychoactive substances except alcohol, including traditional drugs and new psychoactive substances; or smoking >30 cigarettes per day;

(3)Patient with severe disease of heart, liver, spleen, lung, or kidney;

(4)Patient with syphilis or Acquired Immunodeficiency Syndrome (AIDS);

(5)Complicated by severe digestive system diseases and severe malnutrition;

(6)Patient with severe primary diseases of the hematopoietic system;

(7)Patient with abnormal coagulation function;

(8)Those with inflammation, scars, or trauma at the operation site, or those with severe systemic infection;

(9)Those who have a cognitive impairment, cannot cooperate, or are receiving other treatments that may affect the evaluation of the efficacy of this intervention;

(10)Those received any acupuncture treatment in the past six months.

**4. What are the treatments used in this study?**

JTN Therapy will be applied as the intervention in this study. JTN was set up in the name of Professor Jin (Rui Jin), a famous old Chinese medicine expert in China, and is known as the "New School of Lingnan Acupuncture". This therapy has been used in the clinical treatment of various diseases and has shown remarkable effects. Healing the mental dysfunction is not only the main feature of JTN but also the biggest feature of traditional acupuncture. The most commonly used acupoint groups are: Sishen-zhen, Dingshen-zhen, and Shouzhi-zhen, which can gather, restore, flat, and release respectively the dispersed, lost, hyperactive, hypoactive mind and energy.

(1) In the acupuncture group, acupuncture will be added to the drug replacement therapy, with disposable sterile acupuncture needles selected: specifications Φ 0.3×40 mm (1.5 cun), Φ 0.25×25 mm (1 cun). (2) In the sham acupuncture group, sham acupuncture will be added to the treatment of drug replacement therapy.

**5. What will you need to do if participating in this study?**

This study will be recruited through the resident doctors of the Department of Substance Dependence of Guangzhou Hui'ai Hospital during the diagnosis and treatment process, if participating in this study, after signing the informed consent form, you will enter the screening period, and need to receive relevant inspections to determine whether you can participate in this study:

(1) Situations related to psychoactive substance abuse: the type of substance used, time, the way of administration, the frequency of substance used, the dose and time of the last substance use, the number of withdrawal times, Alcohol Use Disorder Identification Test (AUDIT), Michigan Alcohol Screening Test (MAST), etc.;

(2) Auxiliary inspections: blood and urine routine, liver and kidney function, electrocardiogram, etc.

(3) Assessment measurements: alcohol craving degree (VAS and AUQ), Clinical Institute Withdrawal Assessment of Alcohol Scale, Revised (CIWA-Ar), Baker anxiety scale (BAI), drug replacement dosage, Baker depression scale (BDI), Pittsburgh sleep quality index (PSQI), Mental fatigue scale (MFS), General Self-efficacy Scale (GSES), Gastrointestinal symptom rating scale (GSRS), Quality of Life Scale for Patients with Drug Addiction/Dependence (QOL-DA), and relapse, Timeline Followback (TLFB), etc.

(4)Stool collection for analyses of the gut microbiome and the stool metabolomics.

(5) Observation time point: baseline, end of the last intervention (day 14), follow-up for three months. CIWA-Ar and alcohol withdrawal craving (abs-VAS) will also be recorded and evaluated during the treatment.

(6) If your doctor believes that you meet the inclusion and are suitable to participate in it, you will be determined what intervention you will receive based on random numbers generated by the computer, with a 50% chance of entering the acupuncture group or the sham acupuncture group. If you enter the latter, you will receive the conventional treatment (replacement drugs) + sham acupuncture.

The treatment duration is 2 weeks, and the specific treatment regimens of both groups are as follows:

Acupuncture group:

(1) Replacement drugs will be applied as the basic treatment.

(2) Needles: disposable stainless steel sterile acupoint needles (Huatuo brand, Suzhou Medical Supplies Factory Co., Ltd.), specifications Φ 0.3×40mm (1.5 cun), Φ 0.25×25mm (1 cun).

(3) Acupoints: Sishen-zhen: 1.5 cun from the front, back, left, and right of Baihui (GV20).

Dingshen-zhen: 0.5 cun above Yintang (EX-NH3) and Yangbai (GB14) on both sides.

Shouzhi-zhen: Neiguan (PC6), Shenmen (HT7), Laogong (PC8).

(4) Positioning: the *World Health Organization Standard Acupuncture And Acupuncture Point Positioning* (published by the People's Medical Publishing House in 2010) will be adopted for acupoint positioning as follows:

Baihui (GV20): On the head, 5 B-cun superior to the anterior hairline, on the anterior median line.;

Yintang (EX-NH3): On the head, between the right medial end of the eyebrow and the left one.

Yangbai (GB14): On the head, 1B-cun superior to the eyebrow, directly superior to the center of the pupil.

Neiguan (PC6): On the anterior aspect of the forearm, between the tendons of the palmaris longus and the flexor carpi radialis, 2B-cun proximal to the palmar wrist crease.

Shenmen (HT7): On the anteromedial aspect of the wrist, radial to the flexor carpi ulnaris tendon, on the palmar wrist crease.

Laogong (PC8): On the palm, in the depression between the second and third metacarpal bones, proximal to the metacarpophalangeal joints.

(5) Operation: The supine position will be taken, and after the acupoint is located, the operator's hands and acupoint will be routinely disinfected with 75% ethanol, and the specific operation and insertion depth will be as follows:

Among the points selected, through the corresponding tunnels of the real needling device, Shouzhi-zhen will be inserted at an angle of 45–90° to the participant’s skin, while Dingshen-zhen, and Sishen-zhen inserted at 15° (Figure 4). The needles will be inserted at a depth of 5-30 mm. Then the stimulation to achieve the typical acupuncture sensation of de qi characterized as soreness, numbness, and heaviness will be performed manually. The acupuncture needles will be retained for 30 min and twirled every 10 min during each session. The intervention will be discontinued if the patients suffer from any adverse events, and the acupuncturists can decide on termination.

(6) Course of treatment: acupuncture once a day for the first 3 days from admission, and once every other day thereafter, for a total of 2 weeks, with a three-month follow-up.

Control group: Sham acupuncture on the same acupoints will be added to the usual care as a control. Each blunt needle will be inserted into the mouthless patch of the sham needle device, slightly touching the skin and causing a pinprick-like sensation, actually without skin penetration. The needling directions will be the same as that of the acupuncture group. During the needle retention, needle handles will be only touched without any stimulation by acupuncture manipulation once every 10 min, for a total of 3 touches. The set of the treatment environment, posture, treatment session, frequency, and treatment duration will be all equivalent to the acupuncture group, and the assessment points and the follow-up time points in the control group will match those in the experimental group.

(6) After the end of treatment, blood routine, urine routine, liver function, kidney function, electrocardiogram, fecal omics, etc. will be inspected again, and follow-up will be carried out month by month to determine whether the treatment you receive is safe and effective.

During the observation, please take the medicine according to the doctor's instructions, your doctor will tell you in detail the time, method, and follow-up time, and please do not take other drugs on your own during this period; if necessary, please consult your doctor first.

1. **Possible benefits from participating in the study**

You and our society may or may not benefit directly from this study. This benefit includes the potential for improvement in your condition and the possibility that this study may help further clarify the clinical effect of acupuncture as an adjunctive intervention in AUD, and bring good news to patients with conditions similar to yours.

**7. Possible adverse reactions, risks and discomfort, and inconveniences in participating in the study**

During acupuncture, you may have feelings of suan (aching or soreness), ma (numbness or tingling), zhang (fullness, distention, or pressure), and zhong (heaviness), etc., which are normal reactions to acupuncture. Acupuncture may have adverse reactions, but few and mild: though you may experience dizziness due to physical problems or emotional tension, participants in this study will be conducted to take the recumbent position during treatment to reduce the occurrence of this situation, and once the dizziness happened, you may be relieved by yourself after stopping acupuncture intervention and taking a break; additionally, slight bleeding and hematoma, which are part of the other normal reactions of acupuncture, can disappear after local compression.

In addition, any treatment might not be effective, and the condition may continue to develop due to reasons such as ineffective treatment or other diseases. This treatment risk is that everyone seeking medical attention needs to face; the treatment risk exists, even if you do not participate in this clinical study. During the study period, if a physician or investigator finds that the treatments taken by this study are ineffective, the study will be terminated in favor of other treatments that may be effective.

**8. If you are healthy, can you participate in this study? What is the difference?**

This study intends to collect another 10 feces of male adults whose main physical parameters such as age, height, and weight are matched with the patient group for baseline comparison with the patient groups. Therefore, if you meet the following condition, you can also participate: teetotallers or light social drinkers, with Alcohol Use Disorder Identification Test (AUDIT) ≤7 and normal function of cognition and memory assessed by Mini-mental State Examination (MMSE). But please notice that you will not be assigned to any of the intervention methods, beacuse all you need to do is to provide your stool samples for this study, and only once. This project will pay for the cost of the omics testing of the samples, and you will reveive some appropriate amount of subsidies.

1. **Related fees**

The sponsor research group will pay for omics testing, and acupuncture treatment during your participation in this study. Other treatments and examinations, as well as the cost of switching to other treatments due to the suspension of the study, will not be covered free of charge. In the event of research-related damage, the sponsor will pay you the corresponding medical expenses and compensation.

1. **How your privacy will be protected?**

Your medical records (including research records and physical and chemical examination reports, etc.) will be kept in the hospital as required. Your participation and your data in the study are confidential and the report of the results of the study will not reveal your identity. Representatives of the superior health/drug/research administration, hospital ethics committee, investigators, and sponsors will be allowed to access your medical records to verify clinical research procedures and/or data. We will strictly protect the privacy of your personal medical information within the scope of existing laws.

1. **Important tips**

To ensure the reliability of clinical research, throughout the clinical research process, we hope that you could do the following: (1) do not use other similar treatments or drugs on your own; (2) if any discomfort occurs, during the research process, please report to your supervisor in time.

The treatment in this study is not the only option for your current conditions, and you may discuss it with your doctor before deciding whether to participate in this study.

1. **Can you quit after participating in a study?**

Participation is entirely up to you voluntarily. You may refuse to participate in, or withdraw from the study at any time during the research process, without any reason, without any discrimination, retaliation, and destroyment of your medical relationship or interest at all, and you can continue to receive treatment from your doctor.

Your doctor may discontinue your participation in this study at any time out of your best interests.

If you do not participate in this study or quit halfway, there are still many other alternative treatments, so you don't have to participate in this study to treat your illness. If you withdraw from research for any reason, you may be asked about your drug use, and you may also be asked for laboratory and physical inspections if your doctor considers them necessary.

If you choose to participate in this study after full consideration, you will be hoped to consistently complete the entire research process.

1. **Access to more information**

You can ask any questions about this study at any time and your doctor will leave you contact details so that he/she can answer your questions.

If there is any important new information during the course that may affect your willingness to continue with the study, your doctor will notify you on time.

1. **What to do now?**

It is up to you to decide whether or not to participate in this study. You can also discuss it with your family before making a decision. Before deciding to participate in this study, please clear your confusion with your doctor’ s help until you fully understand the study.

Finally, thank you for reading the above. Please carefully confirm whether the above conditions exist. If there are no contraindications above, and you agree to receive relevant inspections, please complete the subject statement. If you decide to participate in this study, tell your doctor and they will arrange everything for you to do about the study. Please retain this material. If you have any questions about your rights and interests in this study, you can contact the Ethics Committee of the Center at **020-81268229** or **020-81268220;** email: **76706230@qq.com**; fax: **020-81580047**.

**Subject Statement**

I have read this informed consent form carefully, the researcher has fully explained the purpose, the operation process, the possible risks and potential benefits of this study, and all my related issues have been answered satisfactorily. I understand that participation is voluntary and that I can choose not to participate in it, or withdraw at any time after notifying the investigator without discrimination or retaliation, and any medical treatment and rights of mine will not be affected as a result.

The researchers may terminate my continued participation in this study if I require additional diagnosis/treatment, if I do not comply with the trial plan, or for other reasonable reasons.

I voluntarily agree to participate in this clinical study and I will receive an original signed "Informed Consent".

Subject's signature: Date: YYMMDD

Contact Number:

Signature of Legal Guardian [if applicable]: Contact Number:

Relation to Subject: Date: YYMMDD

**Researcher Declaration**

I have accurately informed the subject of the informed consent form and answered the questions of the subject, and the subjects volunteered to participate in this clinical study.

Investigator Signature: Date: YYMMDD

Contact Number:
