# Supplementary material for: A protocol for the integration of multi-omics bioinformatics: Mechanism of acupuncture as an adjunctive therapy for alcohol use disorder
Source: Front Neurol. 2023 Jan 5;13:977487. doi: 10.3389/fneur.2022.977487 (PMC9849375; doi:10.3389/fneur.2022.977487)
Supplement: Supplementary file 3 [file Data_Sheet_3.docx]

A Protocol for [the Integration of Multi-omics Bioinformatics Mechanism of Acupuncture as an Adjunctive Therapy for Alcohol Use Disorder](http://www.chictr.org.cn/edit.aspx?pid=164634&htm=4)

Relavant Scales

**Content**

[1 AUDIT questionnaire 3](#_Toc21804)

[2 Michigan Alcohol Screening Test (MAST) 5](#_Toc10276)

[3 Alcohol Urge Questionnaire (AUQ) 7](#_Toc14932)

[4 Visual Analogue-rating scale (VAS) 8](#_Toc25065)

[5 Clinical Institute Alcohol Withdrawal Syndrome Scale (CIWA-Ar) 8](#_Toc4717)

[6 Beck Depression Inventory II (BDI-II) 11](#_Toc1546)

[7 Beck Anxiety Inventory (BAI) 13](#_Toc27365)

[8 Minimum Mental State Examination (MMSE) 14](#_Toc20400)

[9 General Self-Efficacy Scale (GSES) 17](#_Toc12271)

[10 Quality of Life for Drug Addicts (QOL-DA) 17](#_Toc18752)

[11 Gastrointestinal symptom rating scale (GSRS) 19](#_Toc23890)

[12 Mental fatigue scale (MFS) 23](#_Toc5754)

[13 Pittsburgh sleep quality index (PSQI) 28](#_Toc2718)

**1 AUDIT questionnaire[1]**

Please circle the answer that is correct for you

1. How often do you have a drink containing alcohol?

· Never

· Monthly or less

· 2-4 times a month

· 2-3 times a week

· 4 or more times a week

2. How many standard drinks containing alcohol do you have on a typical day when drinking?

· 1 or 2

· 3 or 4

· 5 or 6

· 7 to 9

· 10 or more

3. How often do you have six or more drinks on one occasion?

· Never

· Less than monthly

· Monthly

· Weekly

· Daily or almost daily

4. During the past year, how often have you found that you were not able to stop drinking once you had started?

· Never

· Less than monthly

· Monthly

· Weekly

· Daily or almost daily

5. During the past year, how often have you failed to do what was normally expected of you because of drinking?

· Never

· Less than monthly

· Monthly

· Weekly

· Daily or almost daily

6. During the past year, how often have you needed a drink in the morning to get yourself going after a heavy drinking session?

· Never

· Less than monthly

· Monthly

· Weekly

· Daily or almost daily

7. During the past year, how often have you had a feeling of guilt or remorse after drinking?

· Never

· Less than monthly

· Monthly

· Weekly

· Daily or almost daily

8. During the past year, have you been unable to remember what happened the night before because you had been drinking?

· Never

· Less than monthly

· Monthly

· Weekly

· Daily or almost daily

9. Have you or someone else been injured as a result of your drinking?

· No

· Yes, but not in the past year

· Yes, during the past year

10. Has a relative or friend, doctor or other health worker been concerned about your drinking or suggested you cut down?

· No

· Yes, but not in the past year

· Yes, during the past year

Scoring the AUDIT: Scores for each question range from 0 to 4, with the first response for each question (eg never) scoring 0, the second (eg less than monthly) scoring 1, the third (eg monthly) scoring 2, the fourth (eg weekly) scoring 3, and the last response (eg. daily or almost daily) scoring 4. For questions 9 and 10, which only have three responses, the scoring is 0, 2 and 4 (from left to right).

| **Score interval** | **Indication** | **Coping** |
| --- | --- | --- |
| 0-7 | Alcohol consumption risk level Zone I: no or mild alcohol problems | Alcohol use education is need. |
| 8-15 | Alcohol consumption risk level zone II: moderate alcohol problems | Provision of simple advice on reducing risky drinking is need. |
| 16-19 | Alcohol consumption risk level zone III: high-level of alcohol problems | Recommendation of brief counselling and continued monitoring is need. |
| ≥20 | Alcohol consumption risk level Zone IV: people with suspected alcohol dependence | Further diagnostic assessment and treatment of alcohol dependence is necessary. |

**2 Michigan Alcohol Screening Test (MAST)[2-3]**

| **Item** | **Item content** | **Point for weighted score** | **Point for unit raw score** |
| --- | --- | --- | --- |
| 0 | Do you feel you are a normal drinker? | Not scored | Not scored |
| 1 | Do you feel you drink less than or as much as most other people* | Yes = 0, No = 2 | Yes = 0, No = 1 |
| 2 | Have you ever awakened the morning after some drinking the night before and found that you could not remember a part of the evening? | Yes = 2, No = 0 | Yes = 1, No = 0 |
| 3 | Does your partner, a parent, or other near relative ever worry or complain about your drinking? | Yes = 1, No = 0 | Yes = 1, No = 0 |
| 4 | Can you stop drinking without a struggle after one or two drinks?* | Yes = 0, No = 2 | Yes = 0, No = 1 |
| 5 | Do you ever feel guilty about your drinking? | Yes = 1, No = 0 | Yes = 1, No = 0 |
| 6 | Do friends or relatives think you are a normal drinker?* | Yes = 0, No = 2 | Yes = 0, No = 1 |
| 7 | Are you able to stop drinking when you want to?* | Yes = 0, No = 2 | Yes = 0, No = 1 |
| 8 | Have you ever attended a meeting of Alcoholics Anonymous (AA) because of your own drinking? | Yes = 5, No = 0 | Yes = 1, No = 0 |
| 9 | Have you ever gotten into physical fights when drinking? | Yes = 1, No = 0 | Yes = 1, No = 0 |
| 10 | Has your drinking ever created problems between you and your partner, a parent, or other near relative? | Yes = 2, No = 0 | Yes = 1, No = 0 |
| 11 | Has your partner, a parent, or other near relative ever gone to anyone for help about your drinking? | Yes = 2, No = 0 | Yes = 1, No = 0 |
| 12 | Have you ever lost friends or partners because of your drinking? | Yes = 2, No = 0 | Yes = 1, No = 0 |
| 13 | Have you ever gotten into trouble at work or school because of your drinking? | Yes = 2, No = 0 | Yes = 1, No = 0 |
| 14 | Have you ever lost a job because of your drinking? | Yes = 2, No = 0 | Yes = 1, No = 0 |
| 15 | Have you ever neglected your obligations, your family, or your work for two or more days in a row because you were drinking? | Yes = 2, No = 0 | Yes = 1, No = 0 |
| 16 | Do you drink before noon fairly often? | Yes = 1, No = 0 | Yes = 1, No = 0 |
| 17 | Have you ever been told you have liver trouble? Cirrhosis? | Yes = 2, No = 0 | Yes = 1, No = 0 |
| 18a | After heavy drinking, have you ever had delirium tremens (DTs) or severe shaking, or heard voices or seen things that weren't really there? | With DTs:  No = 0, Yes = 5  Without DTs:  No = 0, Yes = 2 | Yes = 1, No = 0 |
| 18b | (If 18a = Yes) How many times? | Times:_______  Not scored | Times:_______  Not scored |
| **19 | Have you ever gone to anyone for help about your drinking? | Yes = 2, No = 0 | Yes = 1, No = 0 |
| **20 | Have you ever been in a hospital because of your drinking? | Yes = 2, No = 0 | Yes = 1, No = 0 |
| **21 | Have you ever been a patient in a psychiatric hospital or on a psychiatric ward of a general hospital where your drinking was part of the problem that resulted in hospitalization? | Yes = 2, No = 0 | Yes = 1, No = 0 |
| **22 | Have you ever been seen at a psychiatric or mental health clinic or gone to any doctor, social worker, or clergy member for help with any emotional problem where your drinking was part of the problem? | Yes = 2, No = 0 | Yes = 1, No = 0 |
| 23a | Have you ever been arrested for drunken driving, driving while intoxicated, or driving under the influence of alcoholic beverages? | No = 0, Yes = 2 | Yes = 1, No = 0 |
| 23b | (If 23a = Yes) How many times? | Times:_______  Not scored | Times:_______  Not scored |
| 24a | Have you ever been arrested, even for a few hours, because of other drunken behavior? | No = 0, Yes = 2 | Yes = 1, No = 0 |
| 24b | (If 24a = Yes) How many times? | Times:_______  Not scored | Times:_______  Not scored |

*Indicates item is reverse scored.

**This visit to the hospital is not counted in.

The score of Item 24 = 0 or 2*Times

The scale is based on the format of PAR Staff and adopts the revised version of Lingjiang Li.

**3 Alcohol Urge Questionnaire (AUQ)[4]**

Listed below are questions that ask about your feelings about drinking. The words “drinking” and “have a drink” refer to having a drink containing alcohol, such as beer, wine, or liquor. Please indicate how much you agree or disagree with each of the following statements by placing a single mark (like this : X : :) along each line between STRONGLY DISAGREE and STRONGLY AGREE. The closer you place your mark to one end or the other indicates the strength of your disagreement or agreement. Please complete every item. We are interested in how you are thinking or feeling right now as you are filling out the questionnaire.

1. All I want to do now is have a drink.

STRONGLY DISAGREE: ____:____:____:____:____:____:_____: STRONGLY AGREE

2. I do not need to have a drink now.

STRONGLY DISAGREE: ____:____:____:____:____:____:_____: STRONGLY AGREE

3. It would be difficult to turn down a drink this minute.

STRONGLY DISAGREE: ____:____:____:____:____:____:_____: STRONGLY AGREE

4. Having a drink now would make things seem just perfect.

STRONGLY DISAGREE: ____:____:____:____:____:____:_____: STRONGLY AGREE

5. I want a drink so bad I can almost taste it.

STRONGLY DISAGREE: ____:____:____:____:____:____:_____:STRONGLY AGREE

6. Nothing would be better than having a drink right now.

STRONGLY DISAGREE: ____:____:____:____:____:____:_____: STRONGLY AGREE

7. If I had the chance to have a drink, I don’t think I would drink it.

STRONGLY DISAGREE: ____:____:____:____:____:____:_____: STRONGLYAGREE

8. I crave a drink right now.

STRONGLY DISAGREE: ____:____:____:____:____:____:_____: STRONGLY AGREE

**4 Visual Analogue-rating scale (VAS)[5]**

The following 100mm gauge represents the thirst for alcohol. Please circle "⚪" in the corresponding position of the ruler according to your first reaction to your thirst for alcohol, and fill in the value into the space.

Withdrawal craving:

I do not want to drink at all I extremely want to drink

0 10 20 30 40 50 60 70 80 90 100 mm

Cue-related craving:

I do not want to drink at all I extremely want to drink

0 10 20 30 40 50 60 70 80 90 100 mm

Score of quantization (0-100 mm)：withdrawal craving________mm, cue-related craving ________mm

**5 Clinical Institute Alcohol Withdrawal Syndrome Scale (CIWA-Ar)[6]**

Patient name: Gender: Age: Hospitalization Number:

Blood pressure: Heart rate: Evaluation time: Assessors:

| **Order number** | **Item** | | **Score** |
| --- | --- | --- | --- |
| 1 | NAUSEA AND VOMITING  Ask "Do you feel sick to your  stomach? Have you vomited?" Observation. | 0-no nausea and no vomiting  1-mild nausea with no vomiting  4-intermittent nausea with dry heaves  7-constant nausea, frequent dry heaves and vomiting | □ |
| 2 | TREMOR  Arms extended and fingers spread apart. Observation. | 0-no tremor  1-not visible, but can be felt fingertip to fingertip  4-moderate, with patient's arms extended  7-severe, even with arms not extended | □ |
| 3 | PAROXYSMAL SWEATS  Observation. | 0-no sweat visible  1-barely perceptible sweating, palms moist  4-beads of sweat obvious on forehead  7-drenching sweats | □ |
| 4 | TACTILE DISTURBANCES  Ask "Have you any itching, pins  and needles sensations, any burning, any numbness or do you feel  bugs crawling on or under your skin?"  Observation. | 0-none  1-very mild itching, pins and needles, burning or numbness  2-mild itching, pins and needles, burning or numbness  3-moderate itching, pins and needles, burning or numbness  4-moderately severe hallucinations  5-severe hallucinations  6-extremely severe hallucinations  7-continuous hallucinations | □ |
| 5 | AUDITORY DISTURBANCES  Ask "Are you more aware of  sounds around you? Are they harsh? Do they frighten you? Are you  hearing anything that is disturbing to you? Are you hearing things  you know are not there?"  Observation. | 0-not present  1-very mild harshness or ability to frighten  2-mild harshness or ability to frighten  3-moderate harshness or ability to frighten  4-moderately severe hallucinations  5-severe hallucinations  6-extremely severe hallucinations  7-continuous hallucinations | □ |
| 6 | VISUAL DISTURBANCES  Ask "Does the light appear to be  too bright? Is its colour different? Does it hurt your eyes? Are you  seeing anything that is disturbing to you? Are you seeing things you  know are not there?"  Observation. | 0-not present  1-very mild sensitivity  2-mild sensitivity  3-moderate sensitivity  4-moderately severe hallucinations  5-severe hallucinations  6-extremely severe hallucinations  7-continuous hallucinations | □ |
| 7 | ANXIETY  Ask "Do you feel nervous?"  Observation. | 0-no anxiety, at ease  1-mildly anxious  4-moderately anxious, or guarded, so anxiety is inferred  7-equivalent to acute panic states as seen in severe delirium or acute schizophrenic reactions | □ |
| 8 | AGITATION  Observation. | 0-normal activity  1-somewhat more than normal activity  4-moderately fidgety and restless  7-paces back and forth during most of the interview, or constantly thrashes about | □ |
| 9 | HEADACHE, FULLNESS IN HEAD  Ask "Does your head feel  different? Does it feel like there is a band around your head?" Do  not rate for dizziness or lightheadedness. Otherwise, rate severity. | 0-not present  1-very mild  2-mild  3-moderate  4-moderately severe  5-severe  6-very severe  7-extremely severe | □ |
| 10 | ORIENTATION AND CLOUDING OF SENSORIUM  Ask  "What day is this? Where are you? Who am I?"  Observation. | 0-oriented and can do serial additions  1-cannot do serial additions or is uncertain about date  2-disoriented for date by no more than 2 calendar days  3-disoriented for date by more than 2 calendar days  4-disoriented for place and/or person | □ |

Total CIWA-A Score___________

Rater's Initials___________

**6 Beck Depression Inventory II (BDI-II)[7]**

| A | Mood | B | Pessimism |
| --- | --- | --- | --- |
|  | 0=I do not feel sad |  | 0=I am not particularly pessimistic or discouraged about the future |
|  | 1=I feel blue or sad |  | 1=I feel discouraged about the future |
|  | 2=I am blue or sad all the time |  | 2=I feel I have nothing to look forward to |
|  | 3=I am so sad or unhappy that I can’ t stand it |  | 3=I feel that the future is hopeless and that things cannot improve |
| C | Sense of Failure | D | Lack of Satisfaction |
|  | 0=I do not feel like a failure |  | 0=I am not particularly dissatisfied |
|  | 1=I feel I have failed more than the average person |  | 1=I don't enjoy things the way I used to |
|  | 2=As I look back on my life all I can see is a lot of failures |  | 2=I don't get satisfaction out of anything any more |
|  | 3=I feel I am a complete failure as a person (parent, husband, wife) |  | 3=I am dissatisfied with everything |
| E | Guilty Feeling | F | Sense of Punishment |
|  | 0=I have no particular guilt. |  | 0=I don't feel I am being punished |
|  | 1=I feel guilty about a lot of things I did or should have done but didn't. |  | 1=I have a feeling that something bad may happen to me |
|  | 2=I feel guilty most of the time. |  | 2=I feel I am being punished or will be punished |
|  | 3=I feel guilty at all times. |  | 3=I feel I deserve to be punished |
| G | Self Hate | H | Self Accusations |
|  | 0=I don't feel disappointed in myself |  | 0=I don't feel I am any worse than anybody else |
|  | 1=I am disappointed in myself |  | 1= |
|  | 2=I am disgusted with myself |  | 2=I am very critical of myself for my weaknesses or mistakes |
|  | 3=I hate myself |  | 3=I blame myself for everything that goes wrong |
| I | Self-punitive Wishes | J | Crying Spells |
|  | 0=I don't have any thoughts of harming myself |  | 0=I don't cry any more than usual |
|  | 1=I have thoughts of harming myself but I would not carry them out |  | 1=I cry more now than I used to |
|  | 2=I feel I would be better off dead |  | 2=I cry all the time now. I can't stop it |
|  | 3=I would kill myself if I could |  | 3=I used to be able to cry but now I can't cry at all even though I want to |
| K | Anxiety | L | Social Withdrawal |
|  | 0 = I am no more anxious now than in the past. |  | 0=I have not lost interest in other people |
|  | 1=I am more anxious now than usual. |  | 1=I am less interested in other people now than I used to be |
|  | 2=I am very restless and have a hard time keeping quiet. |  | 2=I have lost most of my interest in other people and have little feeling for them |
|  | 3 = I am very restless and have to move or do things all the time. |  | 3=I have lost all my interest in other people and don't care about them at all |
| M | Indecisiveness | N | Body Image |
|  | 0=I can make the same decisions now as I used to. |  | 0 = I don't feel like I'm worthless. |
|  | 1=It's harder for me to make decisions now than it was in the past. |  | 1 = I consider myself less valuable or useful than I used to be. |
|  | 2=My decision is much more difficult than before. |  | 2 = I feel that I am less valuable than others. ​ |
|  | 3=I have a hard time making any decision. |  | 3 = I feel worthless. |
| O | Energy | P | Sleep Disturbance |
|  | 0 = I have the same energy as I used to. |  | 0=I have the same energy as before. |
|  | 1=I don't have as much energy as I used to. |  | 1=I'm not as energetic as I used to be. |
|  | 2=I don't have the energy to do many things. |  | 2=I don't have the energy to do a lot of things. |
|  | 3=I don't have enough energy to do anything. |  | 3=I don't have enough energy to do anything. |
| Q | Irritability | R | Loss of Appetite |
|  | 0=I am no more irritated now than I ever am |  | 0=My appetite is the same as usual. |
|  | 1=I get annoyed or irritated more easily than I used to |  | 1=My appetite is slightly worse than before, or slightly better. |
|  | 2=I feel irritated all the time |  | 2.My appetite is much worse than it used to be, or much better. |
|  | 3=I don't get irritated at all at the things that used to irritate me |  | 3=I have no appetite at all or am always craving food. |
| S | Concentration | T | Fatigability |
|  | 0 = I can concentrate as well as ever. |  | 0=I don't feel tired or tired than I used to be. |
|  | 1=I can't concentrate like I used to. |  | 1=I'm more tired or tired than I used to be. |
|  | 2=I have a hard time concentrating on anything for long periods of time. |  | 2=Because of being too tired or too tired, many things that I used to do can't be done. |
|  | 3=I can't concentrate on anything. |  | 3=Most of the things I used to do can't be done because I'm too tired or too tired. |
| U | Loss of Libido | V | Sum: ______________ |
|  | 0=I have not noticed any recent change in my interest in sex | Note: 0-13=No depression  14-19=Mild depression  20-28=Moderate depression  29-63=Severe depression | |
|  | 1=I am less interested in sex than I used to be |  |  |
|  | 2=I am much less interested in sex now |  |  |
|  | 3=I have lost interest in sex  completely |  |  |

**7 Beck Anxiety Inventory (BAI)[8]**

Below is a list of common symptoms of anxiety. Please carefully read each item in the list. Indicate how much you have been bothered by that symptom during the past month, including today, by circling the number in the corresponding space in the column next to each symptom.

| Item | Not At All - Mildly but it didn’t bother me much - Moderately - it wasn’t pleasant at times - Severely – it bothered me a lot | Selection |
| --- | --- | --- |
| 1.Numbness or tingling | 1—2—3—4 | （ ） |
| 2.Feeling hot | 1—2—3—4 | （ ） |
| 3.Wobbliness in legs | 1—2—3—4 | （ ） |
| 4.Unable to relax | 1—2—3—4 | （ ） |
| 5.Fear of worst happening | 1—2—3—4 | （ ） |
| 6.Dizzy or lightheaded | 1—2—3—4 | （ ） |
| 7.Heart pounding/racing | 1—2—3—4 | （ ） |
| 8.Unsteady | 1—2—3—4 | （ ） |
| 9.Terrified or afraid | 1—2—3—4 | （ ） |
| 10.Nervous | 1—2—3—4 | （ ） |
| 11.Feeling of choking | 1—2—3—4 | （ ） |
| 12.Hands trembling | 1—2—3—4 | （ ） |
| 13.Shaky / unsteady | 1—2—3—4 | （ ） |
| 14.Fear of losing control | 1—2—3—4 | （ ） |
| 15.Difficulty in breathing | 1—2—3—4 | （ ） |
| 16.Fear of dying | 1—2—3—4 | （ ） |
| 17.Scared | 1—2—3—4 | （ ） |
| 18.Indigestion | 1—2—3—4 | （ ） |
| 19.Faint / lightheaded | 1—2—3—4 | （ ） |
| 20.Face flushed | 1—2—3—4 | （ ） |
| 21.Hot/cold sweats | 1—2—3—4 | （ ） |
| Sum：____________ | | |

**8 Minimum Mental State Examination (MMSE)[9]**

| I'm going to start by asking questions that require concentration and memory. Some questions are more dficult that others and some will be asked more than one time.  □(l)What is the year?  □(2)What is the season?  □(3)What is the month?  □(4)What is the date today?  □(5)What Day of the Week Is it?  □(6)What city are we in?  □(7)What district are we in?  □(8)What hospital/street are we in?  □(9)What floor of the building are we on?  □(10)What Is the Name of this Place?  (any appropriate answer all nght, for instance my home, street address, heart study..max score=l)  Now I tell you the names of three things, and please repeat it after I have said it. Please remember them, and I will ask you again in a while (Please repeat it clear. One second for each thing).  These three things are: "tree", "clock", "car". Please repeat.  □tree  □clock  □car  3．Now please do the math, subtract 7 from 100, then count down from the resulting number, and please tell me the answer after each 7 is subtracted until I say "stop".  □100 - 7 =?  □ -7 again =?  □ -7 again =?  □ -7 again =?  □ -7 again =?  4．Now please tell me which three things I made you remember just now?  □tree  □clock  □car  5．The examiner showed the watch and asked the patient what this was?  □1=able to speak correctly  □0=not able to speak correctly  The examiner presented a pencil and asked the patient what this was?  □1=able to speak correctly  □0=not able to speak correctly  6．Please repeat the following:"not Ifs, Ands and Buts"  □1=able to speak correctly  □0=not able to speak correctly  7．(The examiner gives the subject a card that says "Please close your eyes") Please read this sentence and do what it says.  □1=able to speak and do it correctly  □0=not able to speak or do it correctly  8．I will give you a piece of paper, please do as I say:  □Holding the paper in the right hand;  □Fold it in half with both hands;  □Put it on your left leg.  9．Please write me a complete sentence  □1=able to write correctly  □0=not able to write correctly  Write here:      10．Please draw it according to the pattern below.  □1 or 0  The pattern: Draw here: |
| --- |

**9 General Self-Efficacy Scale (GSES)[10]**

| Items | 1 | 2 | 3 | 4 |
| --- | --- | --- | --- | --- |
| 1. I can always manage to solve difficult problems if I try hard enough. | □ | □ | □ | □ |
| 1. If someone opposes me. I can find means and ways to get what I want. | □ | □ | □ | □ |
| 1. It is easy for me to stick to my aims and accomplish my goals. | □ | □ | □ | □ |
| 1. I am confident that I could deal efficiently with unexpected events. | □ | □ | □ | □ |
| 1. Thanks to my resourcefulness, I know how to handle unforeseen situations. | □ | □ | □ | □ |
| 1. I can solve most problems if I invest the necessary effort. | □ | □ | □ | □ |
| 1. I can remain calm when facing difficulties because I can rely on my coping abilities. | □ | □ | □ | □ |
| 1. When I am confronted with a problem, I can usually find several solutions. | □ | □ | □ | □ |
| 1. If I am in a bind, 1 can usually think of something to do. | □ | □ | □ | □ |
| 1. No matter what comes my way, I’m usually able to handle it. | □ | □ | □ | □ |
| Sum：_______________ | | | | |

**10 Quality of Life for Drug Addicts (QOL-DA)[11]**

Dear patient,

First of all, I would like to express my deep sympathy for your suffering from alcohol withdrawal symptoms and my admiration for your determination to give up drinking. As a doctor, we hope to do our best to relieve your pain. Of course, we also need your active cooperation. The following quality of life measurement is to understand your global condition, so as to facilitate doctors to take appropriate treatment and withdrawal measures. This measurement is a popular new method in the world. Just like the nurse constantly measures your temperature with a thermometer, it facilitate doctor to know your global health status. Thanks for your support and cooperate!

Please carefully read each item, and tick one of the five squares according to your actual situation or feeling in the recent week.

| Items | Answer | | | | |
| --- | --- | --- | --- | --- | --- |
|  | No  1 | Mild  2 | Moderate  3 | Serious  4 | Severe  5 |
| 1. Do you feel that you are not as energetic and agile as before? | □ | □ | □ | □ | □ |
| 1. Do you feel weak in some parts of your body? | □ | □ | □ | □ | □ |
| 1. Do you feel dizzy? | □ | □ | □ | □ | □ |
| 1. Has your sexual desire or your interest in the opposite sex diminished? | □ | □ | □ | □ | □ |
| 1. Do you feel a memory loss? | □ | □ | □ | □ | □ |
| 1. Do you feel everything laborious? | □ | □ | □ | □ | □ |
| 1. Do you feel lonely? | □ | □ | □ | □ | □ |
| 1. Are you feeling sad and depressed? | □ | □ | □ | □ | □ |
| 1. Are you disappointed with the future? | □ | □ | □ | □ | □ |
| 1. Do you feel that most people cannot be trusted? | □ | □ | □ | □ | □ |
| 1. Do you feel that you have little value? | □ | □ | □ | □ | □ |
| 1. Do you feel that others ignore you and not sympathize with you? | □ | □ | □ | □ | □ |
| 1. Is your status in the workplace (or at home) affected by drinking alcohol? | □ | □ | □ | □ | □ |
| 1. Do you have to depend on certain substances (wine, medicine) to feel comfortable? | □ | □ | □ | □ | □ |
| 1. Do you feel insecure? | □ | □ | □ | □ | □ |
| 1. Do you feel not free? | □ | □ | □ | □ | □ |
| 1. Do you feel yourself guilty? | □ | □ | □ | □ | □ |
| 1. Did your own drinking bring great pain to your friends and family? | □ | □ | □ | □ | □ |
| 1. Does your own drinking cause serious financial difficulties for your family? | □ | □ | □ | □ | □ |
| Do you have the following symptoms, to what extent? | No | Mild | Moderate | Serious | Severe |
| 1. Running tears or having a runny nose | □ | □ | □ | □ | □ |
| 1. Muscle spasm in the abdomen or elsewhere | □ | □ | □ | □ | □ |
| 1. Calf muscle cramps | □ | □ | □ | □ | □ |
| 1. Waves of cold or fever | □ | □ | □ | □ | □ |
| 1. Nausea or vomiting | □ | □ | □ | □ | □ |
| 1. Diarrhoea | □ | □ | □ | □ | □ |
| 1. Spastic pain in the stomach | □ | □ | □ | □ | □ |
| 1. Perspiration | □ | □ | □ | □ | □ |
| 1. Trembling | □ | □ | □ | □ | □ |
| 1. Difficult breathing | □ | □ | □ | □ | □ |
| 1. Gooseflesh | □ | □ | □ | □ | □ |
|  | Not easy | Little easy | Easy | Very easy | Extremely easy |
| 1. Are you easy to worry? | □ | □ | □ | □ | □ |
| 1. Are you easy to get irritated? | □ | □ | □ | □ | □ |
| 1. Is it easy for you to communicate with others? | □ | □ | □ | □ | □ |
|  | Not | A little | Moderate | Well | Extremely well |
| 1. Can you concentrate? | □ | □ | □ | □ | □ |
| 1. Can you adapt to your surroundings? | □ | □ | □ | □ | □ |
| 1. Can you get any help from your family? | □ | □ | □ | □ | □ |
| 1. Can you get any help from your friends? | □ | □ | □ | □ | □ |
|  | Very bad | Bad | Neither good nor bad | Good | Very good |
| 1. Do you think your family is good? | □ | □ | □ | □ | □ |
| 1. How is your appetite? | □ | □ | □ | □ | □ |
| 1. How are you sleeping? | □ | □ | □ | □ | □ |
| 1. How do you evaluate your overall health status? | □ | □ | □ | □ | □ |
| Sum |  | | | | |

**11 Gastrointestinal symptom rating scale (GSRS)[12]**

This survey contains questions about how you have been feeling and what it has been like DURING THE PAST WEEK. Tick the choice that best applies to you and your situation in the box.

| Items | Answer | | | | | | |
| --- | --- | --- | --- | --- | --- | --- | --- |
|  | No discomfort at all | Minor discomfort | Mild discomfort | Moderate discomfort | Moderately severe discomfort | Severe discomfort | Very severe discomfort |
| 1. Have you been bothered by PAIN OR DISCOMFORT IN YOUR UPPER ABDOMEN OR THE PIT OF YOUR STOMACH during the past week? | □ | □ | □ | □ | □ | □ | □ |
| 1. Have you been bothered by HEARTBURN during the past week? (By heartburn we mean an unpleasant stinging or burning sensation in the chest.) | □ | □ | □ | □ | □ | □ | □ |
| 1. Have you been bothered by ACID REFLUX during the past week? (By acid reflux we mean the sensation of regurgitating small quantities of acid or flow of sour or bitter fluid from the stomach up to the throat.) | □ | □ | □ | □ | □ | □ | □ |
| 1. Have you been bothered by HUNGER PAINS in the stomach during the past week? (This hollow feeling in the stomach is associated with the need to eat between meals.) | □ | □ | □ | □ | □ | □ | □ |
| 1. Have you been bothered by NAUSEA during the past week? (By nausea we mean a feeling of wanting to throw up or vomit.) | □ | □ | □ | □ | □ | □ | □ |
| 1. Have you been bothered by RUMBLING in your stomach during the past week? (Rumbling refers to vibrations or noise in the stomach.) | □ | □ | □ | □ | □ | □ | □ |
| 1. Has your stomach felt BLOATED during the past week? (Feeling bloated refers to swelling often associated with a sensation of gas or air in the stomach.) | □ | □ | □ | □ | □ | □ | □ |
| 1. Have you been bothered by BURPING during the past week? (Burping refers to bringing up air or gas from the stomach via the mouth, often associated with easing a bloated feeling.) | □ | □ | □ | □ | □ | □ | □ |
| 1. Have you been bothered by PASSING GAS OR FLATUS during the past week? (Passing gas or flatus refers to the need to release air or gas from the bowel, often associated with easing a bloated feeling.) | □ | □ | □ | □ | □ | □ | □ |
| 1. Have you been bothered by CONSTIPATION during the past week? (Constipation refers to a reduced ability to empty the bowels.) | □ | □ | □ | □ | □ | □ | □ |
| 1. Have you been bothered by DIARRHEA during the past week? (Diarrhea refers to a too frequent emptying of the bowels.) | □ | □ | □ | □ | □ | □ | □ |
| 1. Have you been bothered by LOOSE STOOLS during the past week? (If your stools (motions) have been alternately hard and loose, this question only refers to the extent you have been bothered by the stools being loose.) | □ | □ | □ | □ | □ | □ | □ |
| 1. Have you been bothered by HARD STOOLS during the past week? (If your stools (motions) have been alternately hard and loose, this question only refers to the extent you have been bothered by the stools being hard.) | □ | □ | □ | □ | □ | □ | □ |
| 1. Have you been bothered by an URGENT NEED TO HAVE A BOWEL MOVEMENT during the past week? (This urgent need to go to the toilet is often associated with a feeling that you are not in full control.) | □ | □ | □ | □ | □ | □ | □ |
| 1. When going to the toilet during the past week, have you had the SENSATION OF NOT COMPLETELY EMPTYING THE BOWELS? (This feeling of incomplete emptying means that you still feel a need to pass more stool despite having   exerted yourself to do so.) | □ | □ | □ | □ | □ | □ | □ |
| Sum |  | | | | | | |

**12 Mental fatigue scale (MFS)[13]**

| Aspect | Items | Answer |
| --- | --- | --- |
| 1. Fatigue | Have you felt fatigued during the past month?  It does not matter if the fatigue is physical (muscular) or mental. If you recently experienced something unusual (for example an accident or short illness) you should try to disregard it when assessing your fatigue. | 0 I do not feel fatigued at all. (No abnormal fatigue, do not need to rest more than usual).  0.5  1 I feel fatigued several times every day but I feel  more alert after a rest.  1.5  2 I feel fatigued for most of the day and taking a  rest has little or no effect.  2.5  3 I feel fatigued all the time and taking a rest  makes no difference. |
| 1. Lack of initiative | Do you find it difficult to start things? Do you experience resistance or a lack of initiative when you have to start something, no matter whether it is a new task or part of your everyday activities? | 0 I have no difficulty starting things.  0.5  1 I find it more difficult starting things than I used to. I’d rather do it some other time.  1.5  2 It takes a great effort to start things. This applies to everyday activities such as getting out of bed, washing myself and eating.  2.5  3 I can’t do the simplest of everyday tasks (eating, getting dressed). I need help with everything. |
| 1. Mental fatigue | Does your brain become fatigued quickly when you have to think hard? Do you become mentally fatigued from things such as reading, watching TV or taking part in a conversation with several people?  Do you have to take breaks or change to another activity? | 0 I can manage in the same way as usual. My ability for sustained mental effort is not reduced.  0.5  1 I become fatigued quickly but am still able to make the same mental effort as before.  1.5  2 I become fatigued quickly and have to take a break or do something else more often than before.  2.5  3 I become fatigued so quickly that I can do nothing or have to abandon everything after a short period ( 5 minutes). |
| 1. Mental recovery | If you have to take a break, how long do you need to recover after you have worked ‘until you drop’ or are no longer able to concentrate on what you are doing? | 0 I need to rest for less than an hour before continuing whatever I am doing.  0.5  1 I need to rest for more than an hour but do not require a night’s sleep.  1.5  2 I need a night’s sleep before I can continue whatever I am doing.  2.5  3 I need several days rest in order to recover. |
| 1. Concentration difficulties | Do you find it difficult to gather your thoughts and concentrate? | 0 I can concentrate as usual.  0.5  1 I sometimes lose concentration, for example when reading or watching TV.  1.5  2 I find it so difficult to concentrate that I have problems, for example reading a newspaper or taking part in a conversation with a group of people.  2.5  3 I always have such difficulty concentrating that it is almost impossible to do anything. |
| 1. Memory problems | Do you forget things more often than before, do you need to make notes or do you have to search for things at home or at work? | 0 I have no memory problems.  0.5  1 I forget things slightly more often than I should, but I am able to manage by making notes.  1.5  2 My poor memory causes frequent problems (for example forgetting important meetings or turning off the cooker).  2.5  3 I can hardly remember anything at all. |
| 1. Slowness of thinking | Do you feel slow or sluggish when you think about something? Do you feel that it takes an unusually long time to conclude a train of thought or solve a task that requires mental effort? | 0 My thoughts are neither slow nor sluggish when it comes to work involving mental effort.  0.5  1 My thoughts are a bit slow one or a few times each day when I have to do something that requires serious mental effort.  1.5  2 My thoughts often feel slow and sluggish, even when carrying out everyday activities, for example a conversation with a person or when reading the newspaper.  3 My thoughts always feel very slow and sluggish. |
| 1. Sensitivity to stress | Do you find it difficult to cope with stress, that is doing several things at the same time while under time pressure? | 0 I am able to cope with stress in the same way as usual.  0.5  1 I become more easily stressed, but only in demanding situations that I was previously able to manage.  1.5  2 I become stressed more easily than before. I feel stressed in situations that previously did not bother me.  2.5  3 I become stressed very easily. I feel stressed in unfamiliar or trying situations. |
| 1. Increased tendency to become emotional | Do you find that you cry more easily than previously?  Do you often burst into tears when, for example, you watch a sad film or talk with your family members?  If you recently experienced something unusual  (for example an accident or short illness) you  should try to disregard it in your assessment. | 0 I am not more emotional than I used to be.  0.5  1 I am more emotional than other people but it is something that is natural for me. I start to cry or my eyes fill with tears easily, but only in relation to things that affect me deeply.  1.5  2 My emotions are problematic or embarrassing. I sometimes even start to cry about things that mean nothing to me. I try to avoid certain situations because of this.  2.5  3 My emotions cause me great problems. They disturb my day-to-day relationship with members of my immediate family and make it difficult for me to cope outside the home. |
| 1. Irritability or ‘a short fuse’ | Are you unusually short-tempered or irritable about things that previously did not bother you? | 0 I am not more short-tempered or irritable than I used to be.  0.5  1 I become more easily irritated, but it does not last very long.  1.5  2 I become irritated very quickly about small things or things that do not bother other people.  2.5  3 I react with extreme anger or rage, which I find very difficult to control. |
| 1. Sensitivity to light | Are you sensitive to strong light? | 0 I have no increased sensitivity to light.  0.5  1 I sometimes experience problems with strong light such as sunlight reflected by snow, water or glass or strong lights at home, but I am able to cope with it, for example by wearing sunglasses.  1.5  2 I am so sensitive to light that I prefer to carry out my daily activities in dim light. I find it difficult to leave the house without sunglasses.  2.5  3 My sensitivity to light is so strong that I am unable to leave the house without sunglasses. I keep the blinds (or equivalent) drawn at all times. |
| 1. Sensitivity to noise | Are you sensitive to noise? | 0 I do not suffer from increased sensitivity to noise.  0.5  1 I sometimes have difficulty with loud noise (for example music, noise from the TV or radio or sudden, unexpected sounds), but I can deal with it easily by turning down the volume. My sensitivity to noise does not disturb my everyday life.  1.5  2 I have a marked over-sensitivity to noise. I have to avoid loud noise or reduce it (for example by means of ear plugs) in order to cope with everyday life.  2.5  3 My sensitivity to noise is so great that I find it difficult to manage at home despite sound insulation. |
| 1. Decreased sleep at night | Do you sleep badly at night? If you are sleeping more than before at night, please place a circle around the ‘0’. If you are taking sleeping tablets and sleep normally, please place a circle around the ‘0’. | 0 I do not sleep less than before.  0.5  1 I have slight problems falling asleep or my sleep is shorter, lighter or more restless than before.  1.5  2 I sleep at least 2 hours less than before and wake up frequently during the night without anything disturbing me.  2.5  3 I sleep less than 2–3 hours per night. |
| 1. Increased sleep | Do you sleep longer and/or more deeply than before?  If you are sleeping less than before, please place a circle around the ‘0’. N.B. Please take account of time spent sleeping during the day. | 0 I do not sleep more than usual  0.5  1 I sleep longer or deeper, but less than 2 hours more than usual, including naps during the day.  1.5  2 I sleep longer or deeper. At least 2 hours more than usual, including naps.  2.5  3 I sleep longer or deeper. At least 4 hours more than usual and in addition I need to take a nap during the day. |
| 1. 24-hour variations | Do you find that at certain times of the day or night the problems we asked about (for example tiredness, lack of concentration) are better or worse? In the statements below, ‘regularly’ means at least 3–4 days of the week. | 0 I have not noticed that my problems are regularly better or worse at certain times, or I do not have any specific problems.  1 There is a clear difference between certain times of the day. I can predict that I will feel better at certain times and worse at other times.  2 I feel unwell at all times of the day and night. |
|  | If you experience 24-hour variations: | When do you feel at your best?  Morning Afternoon Evening Night  When do you feel at your worst?  Morning Afternoon Evening Night |
| Sum | |  |

**13 Pittsburgh sleep quality index (PSQI)[14]**

| Items | Answer | | | |
| --- | --- | --- | --- | --- |
|  | 0 | 1 | 2 | 3 |
| 1. During the past week, when have you usually gone to bed at night? | USUAL BED TIME: | | | |
| 2.During the past week, when have you usually gotten up in the morning? | USUAL GETTING UP TIME: | | | |
| 3.During the past week, how many hours of actual sleep did you get at night? (This may be different than the number of hours you spend in bed.)  HOURS OF SLEEP PER NIGHT: | | | | |
| 1. During the past week, how long (in minutes) has it usually take you to fall asleep each night? | □≤15 min | □16-30 min | □31-60 min | □≥60 min |
| 1. During the past week, how often have you had trouble sleeping because you... | | | | |
| a.Cannot get to sleep within 30 minutes | □Not | □＜Once / week | □1-2 times / week | □≥3 times / week |
| b. Wake up in the middle of the night or early morning | □Not | □＜Once / week | □1-2 times / week | □≥3 times / week |
| c. Have to get up to use the bathroom | □Not | □＜Once / week | □1-2 times / week | □≥3 times / week |
| d. Cannot breathe comfortably | □Not | □＜Once / week | □1-2 times / week | □≥3 times / week |
| e. Cough or snore loudly | □Not | □＜Once / week | □1-2 times / week | □≥3 times / week |
| f. Feel too cold | □Not | □＜Once / week | □1-2 times / week | □≥3 times / week |
| g. Feel too hot | □Not | □＜Once / week | □1-2 times / week | □≥3 times / week |
| h. Had bad dreams | □Not | □＜Once / week | □1-2 times / week | □≥3 times / week |
| 1. Have pain | □Not | □＜Once / week | □1-2 times / week | □≥3 times / week |
| j.Other reason(s) | □Not | □＜Once / week | □1-2 times / week | □≥3 times / week |
| please describe: _______________________________________________________________________ | | | | |
| 6.During the past week, how would you rate your sleep quality overall? | □ Very good | □ Fairly good | □ Fairly bad | □ Very bad |
| 7.During the past week, how often have you taken medicine (prescribed or “over the counter”) to help you sleep? | □Not During the past week | □＜Once / Week | □1-2 times / Week | □≥3 times / Week |
| 8.During the past week, how often have you had trouble staying awake while driving, eating meals, or engaging in social activity? | □Not During the past week | □＜Once / Week | □1-2 times / Week | □≥3 times / Week |
| 9.During the past week, how much of a problem has it been for you to keep up enough enthusiasm to get things done? | □No problem at all | □Only a very slight problem | □Somewhat of a problem | □A very big problem |
| Sum: ___________________ | | | | |

**Reference**

1. Babor, T. F., Higgins-Biddle, J. C., Saunders, J. B., Monteiro, M. G. *The alcohol use disorders identification test*. Geneva: World Health Organization; (2001).

2. PAR Staff. Administration and Scoring of the Michigan Alcohoism Screening Test (MAST) (2021). Available from: URL:https://www.parinc.com/Portals/0/Webuploads/samplerpts/ChecKIT%20Series_MAST_Tech%20Supp%20Paper%20(2).pdf

3. Zhang M. *Handbook of psychiatric assessment scales 2nd Edition*. Changsha, Hunan, China: Hunan Science and Technology Press; (1998).

4. Bohn M J, Krahn D D, Staehler B A. Development and initial validation of a measure of drinking urges in abstinent alcoholics. *Alcoholism: clinical and experimental research* (1995) 19(3): 600-606.

5. Wewers M E, Lowe N K. A critical review of visual analogue scales in the measurement of clinical phenomena. *Research in nursing & health* (1990) 13(4): 227-236.

6. Sullivan J T, Sykora K, Schneiderman J, Naranjo C A, Sellers E M. Assessment of alcohol withdrawal: the revised clinical institute withdrawal assessment for alcohol scale (CIWA‐Ar). *British journal of addiction* (1989) 84(11): 1353-1357.

7. Beck A T, Ward C H, Mendelson M, Mock J, Erbaugh J. An inventory for measuring depression. Archives of general psychiatry (1961) 4(6): 561-571.

8. Beck, A. T., Epstein, N., Brown, G., Steer, R. A. An inventory for measuring clinical anxiety: Psychometric properties. *Journal of Consulting and Clinical Psychology* (1988) 56, 893-897.

9. Mowla A, Zandi T. Mini-mental status examination: a screening instrument for cognitive and mood disorders of elderly. *Alzheimer Disease & Associated Disorders* (2006) 20(2): 124.

10. Schwarzer, R., Bäßler, J., Kwiatek, P., Schröder, K., Zhang, J. X. The assessment of optimistic self‐beliefs: comparison of the German, Spanish, and Chinese versions of the general self‐efficacy scale. *Applied Psychology* (1997) 46(1), 69-88.

11. Wan C, Fang J, Chen L, He L, Gao Y. Development and evaluation of quality of life scale for drug addicts. *Chinese behavioral medicine science* (1997) (03):11-13.

12. Svedlund, J., Sjödin, I., Dotevall, G. GSRS—a clinical rating scale for gastrointestinal symptoms in patients with irritable bowel syndrome and peptic ulcer disease. *Digestive diseases and sciences* (1988) 33(2), 129-134.

13. Johansson B, Starmark A, Berglund P, Rödholm M, Rönnbäck L. A self-assessment questionnaire for mental fatigue and related symptoms after neurological disorders and injuries. *Brain Inj* (2010) 24:2–12. doi: 10.3109/02699050903452961

14. Buysse DJ, Reynolds CF, Monk TH, Berman SR, Kupfer DJ. The Pittsburgh sleep quality index: A new instrument for psychiatric practice and research. *Psychiatry Res* (1989) 28:193–213. doi: https://doi.org/10.1016/0165-1781(89)90047-4
